# A system-level mechanistic explanation for asymmetric stem cell fates: *Arabidopsis thaliana* root niche as a study system

**DOI:** 10.1038/s41598-020-60251-8

**Published:** 2020-02-26

**Authors:** Mónica L. García-Gómez, Diego Ornelas-Ayala, Adriana Garay-Arroyo, Berenice García-Ponce, María de la Paz Sánchez, Elena R. Álvarez-Buylla

**Affiliations:** 1 Departamento de Ecología Funcional, Instituto de Ecología, Universidad Nacional Autónoma de México, Coyoacán, Ciudad de México, México; 2 Centro de Ciencias de la Complejidad, Universidad Nacional Autónoma de México, Coyoacán, Ciudad de México, México; 3 Present Address: School of Life Sciences, University of Warwick, Coventry, United Kingdom

**Keywords:** Stem-cell niche, Gene regulatory networks, Plant molecular biology

## Abstract

Asymmetric divisions maintain long-term stem cell populations while producing new cells that proliferate and then differentiate. Recent reports in animal systems show that divisions of stem cells can be uncoupled from their progeny differentiation, and the outcome of a division could be influenced by microenvironmental signals. But the underlying system-level mechanisms, and whether this dynamics also occur in plant stem cell niches (SCN), remain elusive. This article presents a cell fate regulatory network model that contributes to understanding such mechanism and identify critical cues for cell fate transitions in the root SCN. Novel computational and experimental results show that the transcriptional regulator SHR is critical for the most frequent asymmetric division previously described for quiescent centre stem cells. A multi-scale model of the root tip that simulated each cell’s intracellular regulatory network, and the dynamics of SHR intercellular transport as a cell-cell coupling mechanism, was developed. It revealed that quiescent centre cell divisions produce two identical cells, that may acquire different fates depending on the feedback between SHR’s availability and the state of the regulatory network. Novel experimental data presented here validates our model, which in turn, constitutes the first proposed systemic mechanism for uncoupled SCN cell division and differentiation.

## Introduction

**Figure 1:**
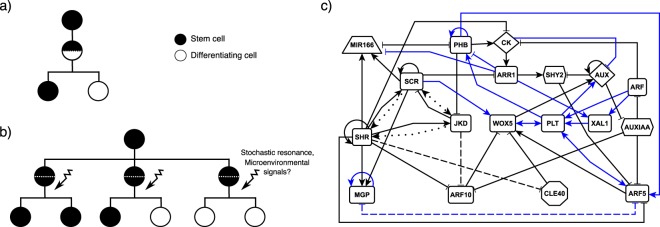
Models of SC asymmetric division: (**a**) SC divisions are invariantly asymmetric, or (**b**) SC divisions are uncoupled from cell differentiation, such that a division produces two identical cells that can later acquire different fates. In this latter scenario, asymmetry can exist at the population level (reviewed in Simons and Clevers, [2011]). (**c**) Regulatory network model of the root SCN^53^. We updated the network and show the newly incorporated interactions (blue).

Stem cells (SCs) are undifferentiated cells that continuously produce the cells necessary to maintain post-embryonic tissues in multicellular organisms^1,2^. Upon cell division, SCs can produce both daughter cells to renew themselves and cells that leave the niche and actively proliferate until differentiating^1,2^. Both the transition from stemness to cell proliferation and the perpetuation of the cells necessary and sufficient for organ maintenance, depend on SCs’ ability to divide asymmetrically^3^. Asymmetry cannot always be understood at the individual cell level (Fig. 1a), as SCs divisions are not always coupled with cell differentiation or with asymmetric characteristics of the daughter cells (Fig. 1b)^4–10^. The population asymmetry model suggests that, in a SC population, certain cell divisions may yield two SCs, others may yield two cells that will differentiate, and yet others may yield one of each (Fig. 1b). The fate of the SC progeny in each case could be defined stochastically^4,5,8^, although restrictions imposed by molecular signals (i.e., short-range niche signals) and space limitations at the niche may also be involved^7,11,12^.

Dynamical models of gene regulatory networks have been used to study cell differentiation and pattern formation in plant and animal development^13–17^. These computational models describe cell types or fates as attractors and have shown that cell-fate decisions emerge from the feedback between intracellular regulatory networks and extracellular signals^13,18^. In these models, the extracellular signals may vary according to the position of a cell within a spatial domain. It is possible that these complex mechanisms could also participate in defining the fate of SCs’ progeny^5,17–25^, where some of the spatial signals could be emanating from the organizer or other niche cells^1,2,26–35^. Dynamical models of gene regulatory networks define an attractor landscape: a multidimensional and non-linear potential that restricts the possible transitions among the network attractors (cell types)^16,36–38^. For spatial signals to influence the differentiation dynamics of SCs, they must impinge on the underlying gene regulatory networks. For example, by affecting the activity of an individual regulator of the intracellular networks, causing a reshaping of the attractor landscape. This reshaping could then facilitate the transition between two particular attractors (cell types or fates)^39–41^. In the context of a SCN, these external signals may provide niche-dependent information and could explain the stereotypical differentiation patterns observed during SC divisions. Symmetric and asymmetric SC divisions could each be the result of the same multistable regulatory mechanism under different spatial conditions within the niche. In this article we hypothesize that a mechanism like this is behind the asymmetric divisions at the root stem cell niche (SCN) of *Arabidopsis thaliana* (“Arabidopsis” herein). We used a complex-systems approach to identify the signals that could be critical for the asymmetric SC divisions in the root SCN, and then studied the cell-fate decisions during SC divisions as a dynamic process resulting from the feedback between the intracellular regulatory network underlying cell fate and an extracellular signal that reshapes the attractor landscape, and hence, cell fate.

**Figure 2:**
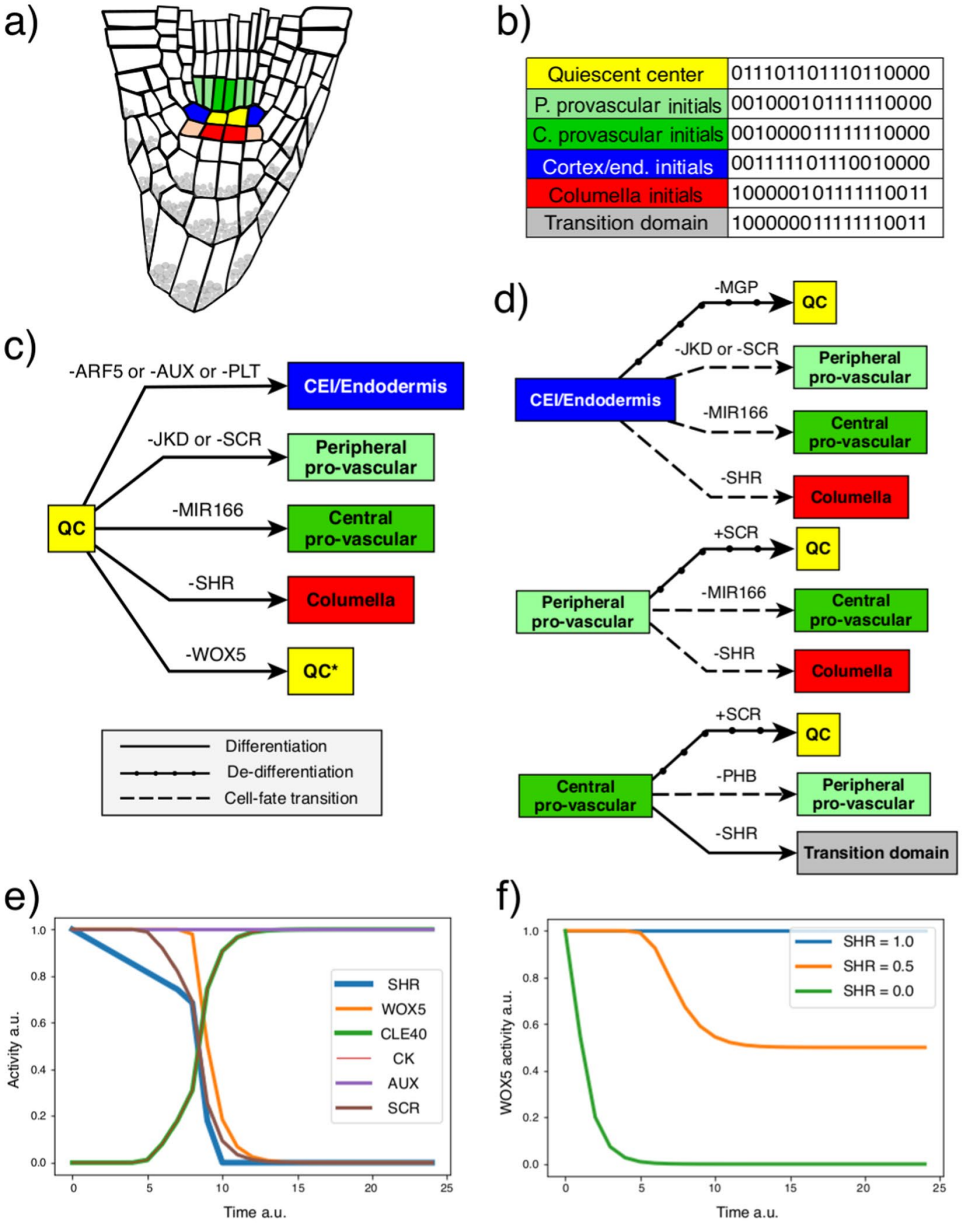
Attractor transitions caused by quantitative variations in the decay rate of the regulators of the network. (**a**) The root SCN consists of the QC cells (yellow) that are surrounded by the cortex/endodermis initials (blue), the pro-vascular initials (green, sub-differentiated into peripheral [P.] and central [C.]), the columella initials (red), and the lateral root cap/epidermis initials (orange). (**b**) We assumed constitutive auxin (AUX) activity. The attractors recovered by the regulatory network model with this condition correspond to the activity profiles of these root SCN cells, and a transition domain attractor that represent cells that exit the meristem and begin to differentiate. The activity of the regulators in the attractors are in the following order: CLE40, WOX5, SHR, SCR, MGP, JKD, MIR166, PHB, XAL1, PLT, ARF, ARF10, ARF5, AUX, AUXIAA, SHY2, CK, and ARR1. (**c**) Transitions from the QC to the initial cells’ attractors. The coloured boxes represent the attractors of the model, while the connecting arrows show the direction of attractor transitions. The regulators on each arrow indicate that its downregulation (−) causes the respective transition. (**d**) Transitions between the rest of the initial cells’ attractors: the transition from the pro-vascular attractors to the QC attractor is caused, in this case, by the upregulation (+) of a regulator. (**e**) Temporal activity of cell-fate regulators in the transition from the QC to the columella initials attractor: SCR and WOX5 were used as markers of the QC cells, and CLE40 and CK as markers of columella initials cells. F) WOX5 activity in the context of different activity levels of SHR. Time and activity are in arbitrary units (a.u.).

The root SCN consists of the quiescent centre (QC) cells and the surrounding initials (Fig. 2a). The QC is the organizer centre of the niche from which short-range signals are produced; these signals maintain the initial cells in an undifferentiated state^27,34^. The initial cells divide asymmetrically, and, depending on their location relative to the QC cells, each type generates progeny committed to assuming the identity of a specific tissue^42^. The QC cells rarely divide in optimal growth conditions at 5 dpg (days post-germination)^43^, making it experimentally challenging to analyse what types of initial cells it is capable of producing^44–46^. Some studies have addressed the mechanisms that regulate the timing of the division of the QC cells^46,47^, but the mechanisms underlying the cell-fate decisions during asymmetric divisions remain unknown. Clonal analyses have shown that the QC cells divide asymmetrically, with one daughter cell renewing the QC while the other becoming either a columella or a cortex/endodermis (CEI) initial cell^46,48^. Indirect evidence suggests that pro-vascular initials can also be produced in rare occasions^49^, but, by far, the most common fate is to produce columella initials^46^. Nonetheless, it is not yet clear what is the underlying mechanism for this biased cellular pattern nor under which conditions the QC could produce the other types of initial cells.

Anticlinal QC cell divisions add cells to the existing cell layer surrounding the pro-vascular tissues, while periclinal divisions create new cell layers^50^. QC cell divisions are mostly periclinal (examples can be found in^45,46,51,52^). Temporal expression dynamics of cell identity markers suggest that every periclinal cell division yields two QC cells that, after several days, acquire different fates^46^. It is reasonable to think that the spatial context in which the QC progeny is found after a periclinal division could be providing molecular cues that guide their posterior fate: to remain as a QC cell or to differentiate into one of the initial cells. To identify such signals we used an updated version of a published mathematical model of the gene regulatory network underlying cell fate decisions at the root SCN (Fig. 1c)^53^. Using this model, we performed a decay-rate variation analysis^39^ to theoretically predict the regulators that might be causing cell-fate transitions within the root SCN. Interestingly, our computational analysis predicted that variations in the activity of SHORTROOT (SHR) within the daughter cells of a QC cell division could explain the biased production of columella initials.

SHR—a GRAS transcriptional regulator—is a key regulator of both endodermal^54,55^ and QC cell fate specification^32^. The pro-vascular tissues are the only place in the root meristem where this regulator’s expression has been observed^55^, and from which SHR’s protein moves through the symplast to the adjacent layer^56^. Importantly, *SHR* is not expressed in the QC cells, but instead these cells receive it from the pro-vascular neighbouring tissues. To explore if the constrained *SHR*’s expression domain, SHR’s regulated mobility, and its feedback with the regulatory network could altogether explain the observed cell fate transitions after QC divisions, we developed a multi-scale model of the root tip. This model allowed us to follow the activity of the intracellular regulatory network in each simulated cell, and to couple cell networks via SHR movement. Interestingly, we found that, depending on the division axis of the QC (and how it constrains SHR availability), both symmetric and asymmetric divisions can take place in the simulation platform. We further used this multi-scale model and an experimental counterpart to study the fate of the QC cells and its progeny upon changes in the range of activity of SHR. Altogether, our model and its experimental validation show that the biased commitment to columella initials observed during asymmetric divisions of the QC cells is the result of the dynamic feedback between the intracellular regulatory network, SHR intercellular movement, SHR intracellular levels, and the cell division plane. The results we present strongly support that the dynamics of SC division at the root SCN follow the population asymmetry model in which a regulatory feedback between signals of the microenvironment and the intracellular regulatory network drive cell fate decisions.

## Results

### Reshaping the attractor landscape of the root SCN regulatory network: SHR is key for the cell fate transition from QC to columella initials

We first introduce the experimentally grounded regulatory network model (Fig. 1c)^53^ that we will study. We previously developed a Boolean model of the regulatory network in the root meristem that integrates well characterized regulatory interactions between transcriptional regulators, and the auxin and cytokinin signaling pathways in the root meristem (the interactions can be found in^53^). Based on an exhaustive literature review, we defined logical functions (one per regulator) that formalize experimental information about the regulation of each element of the network; the logical rules consider regulation at the transcriptional, post-transcriptional and protein activity levels. The logical functions are applied to all possible states of the system (2^n^; where 2 is the two possible states of activity [active or inactive], and n is the number of modelled nodes) to find the state of the system in the next time step. By updating the state of the nodes with the logical rules iteratively, the system eventually converges at activity configurations that do not change anymore, known as attractors. The attractors satisfy the constraints imposed by the logical rules and the system will remain in those states, unless perturbed. The activity configuration of each attractor can be associated with that of different cell types of the experimental system under study, based on documented experimental activity patterns. For example, the Boolean model of the root meristem recovered attractors corresponding to different cell types along the meristem, including those at the root SCN^53^. It is possible to transform the Boolean model to a system of ordinary differential equations to model the activity of the nodes as continuous variables. The continuous model recovers the same attractors as the Boolean network, and this is not dependant on the specific parameters used^53^. The advantage of using a continuous version of the Boolean model is that it is possible to quantitatively vary the parameters and analyse the effect of those changes on the state of the system; namely, to analyse if those perturbations cause attractor transitions. The latter represent a transition from one cell type to another one.

We updated the regulatory network model we previously reported^53^ with new experimental information (Supplementary Table S1); for instance, we included the role of XAL1 and PLT transcription factors (Fig. 1c). The updated regulatory network recovered attractors equivalent to those reported previously^53^. In the present study we aimed to study the cell transitions at the root SCN, where auxin levels are high^57,58^. Hence, we defined the node representing this hormone as constitutively active (AUX = 1). The regulatory network model with this condition recovered attractors representing the following cell types of the root SCN: QC, CEI/Endodermis, peripheral pro-vascular initials, central pro-vascular initials, and columella initials (Fig. 2b).

Then, we used the continuous version of the model and performed a decay rate variation analysis^39^, in order to identify if individual regulators of the network could be mediating attractor transitions (Supplementary Tables C). To perform this analysis, we gradually increased the decay rate of each regulator and analysed the new attractor attained when initiating the system from any other particular attractor. If the activity of the nodes in the new attractor did not correspond to that of the original attractor, that meant that the decay rate alteration caused a transition from one attractor to another. Importantly, we varied the decay rates as a methodological strategy to identify candidates, but functionally their role is not limited to the regulation of their decay rate; instead the candidates could constitute positional information, or could be the targets of developmental, physical, chemical, or environmental cues regulating their expression or protein activity to influence the output of a SC division towards each one of the cell types within the niche. We performed this analysis and found several regulators which, when altered, were sufficient to cause a transition between the attractors of the regulatory network (Fig. 2).

First we will discuss the candidates that could be underlying the asymmetric divisions of the QC cells. We found that decreasing SHR activity caused a transition from the QC to the columella initial attractor; decreasing JACKDAW (JKD) or SCARECROW (SCR) causes a QC cell attractor transition to the peripheral pro-vascular initials attractor; decreasing microRNA165/6 (MIR166) causes a transition to the central pro-vascular initials attractor; and decreasing AUXIN RESPONSE FACTOR 5 (ARF5), PLETHORA (PLT), or AUX cause a transition to the CEI/Endodermis attractor (Fig. 2c). Notably, SHR is the only candidate regulator found through this analysis which alterations may yield a transition from the QC to the columella initials attractor (Fig. 2c), that is the most commonly observed cell transition from the QC cells^46^—. The model allowed us to monitor how the activity of other regulators of the network change as SHR levels decrease (Fig. 2e). This simulates how the network responds during this transition and shows that the candidate regulators predicted, in this case SHR, do not work in isolation, but instead in the context of a network of many interacting elements. We also considered how the QC attractor responded to arbitrary levels of SHR activity. We found that high levels of SHR maintained the QC attractor, intermediate levels of SHR produced a mixed QC-columella initials cell state, and low SHR activity yielded a columella initial cell state (Fig. 2f). Finally, we assessed if the reverse transition was possible; if increasing SHR activity could cause a transition from the columella initials to the QC attractor. We found that SHR activity was insufficient to cause the reverse attractor transition (Supplementary Fig. 3), indicating a directionality in the attractor transitions predicted by our decay-rate variation analysis when modifying SHR levels.

We also found candidate regulators that may be important for explaining the differentiation of QC cells into CEI^48^. The transition from the QC to the CEI/Endodermis attractor is caused by network regulators related to auxin responses (Fig. 2c). This result agrees with the known role of auxin levels in defining the position of the QC in the cells of the layer adjacent to the pro-vasculature^57^, which would otherwise acquire an endodermis fate. Our decay-rate variation analysis correctly predicted this known role of auxin as an important signal for the transition of the QC cells to the endodermal initial cells. The downregulation of the predicted candidate regulators, namely PLT, ARF5 and AUX, may be underlying the reported production of CEI during QC anticlinal cell divisions^48^.

Through our analysis, we also identified regulators that cause transitions among the rest of the attractors (Fig. 2d). In some cases these transitions imply de-differentiation and cell fate transition events in the root meristem. For instance, we found that the CEI/Endodermis attractor transitions to the QC attractor when MAGPIE (MGP) activity decreases, implying the de-differentiation of these initial cells back to the multipotent QC cell state. We also found candidate regulators that can individually mediate the transition from the CEI/Endodermis attractor towards the peripheral and central pro-vascular initials attractors (Fig. 2d), but we did not find candidate regulators for the reverse transition. Clonal analysis had detected this cell fate transition event, and its directionality, as cell layer invasion events in the root meristem^49^. In these events, clones starting in the endodermal or cortex cells divide periclinally, invading the inner tissues of the pro-vasculature. Based on our results, we argue that a downregulation of MIR166, JKD, or SCR activity could explain these experimentally observed cell fate transitions (Fig. 2d).

Some cell-fate transitions described experimentally were not recovered by the decay-rate variation analysis we performed. For example, the pro-vascular cells of the root meristem can regenerate the QC upon the excision of the root apex or upon the ablation of the original QC cells^59–61^. This transition was not recovered by our analysis. It is possible that this cell fate transition is not mediated solely by the change in the activity of a single component of the regulatory network. Alternatively, this transition might be mediated by a more complex alteration derived from the ablation/excision experiments. Nonetheless, to identify some of the critical regulators that could be participating in this attractor transition, we explored the effect of simulating the ectopic activation of regulators that are typically inactive in the peripheral and central pro-vascular attractors (Supplementary Fig. 1). We found that SCR activity—normally not found in the pro-vascular cells of the meristem—is sufficient to cause the transition of the pro-vascular attractors to the QC attractor (Fig. 2d). This result predicts that the ectopic activity of SCR in the pro-vascular cells could be critical for QC regeneration; it would allow SHR to positively regulate its targets in these cells during the extraordinary conditions posed by regeneration.

Overall, our model identified critical regulators of the regulatory network as candidates that may induce cell state transitions in the root SCN. Some of these transitions have been previously observed but no candidate regulators had been suggested to explain them. The predictions of this analysis could inform future experiments to mechanistically explain these cell-fate transitions events, where no underlying mechanism has been proposed. Regarding the QC asymmetric cell division, we predicted regulators that can cause the transition from the QC attractor to each of the initial cells attractors, posing them as potential regulators of QC asymmetric divisions. Particularly, decreasing SHR activity triggers the experimentally described transition from the QC to the columella initials cell fate^46^, suggesting that variations in SHR levels within the progeny of the QC could explain that—in contrast to other cell types—columella initials are preferentially produced by QC cell divisions.

### Development of a multi-scale model of SHR intercellular transport and the dynamic regulatory network in the root stem cell niche

**Figure 3:**
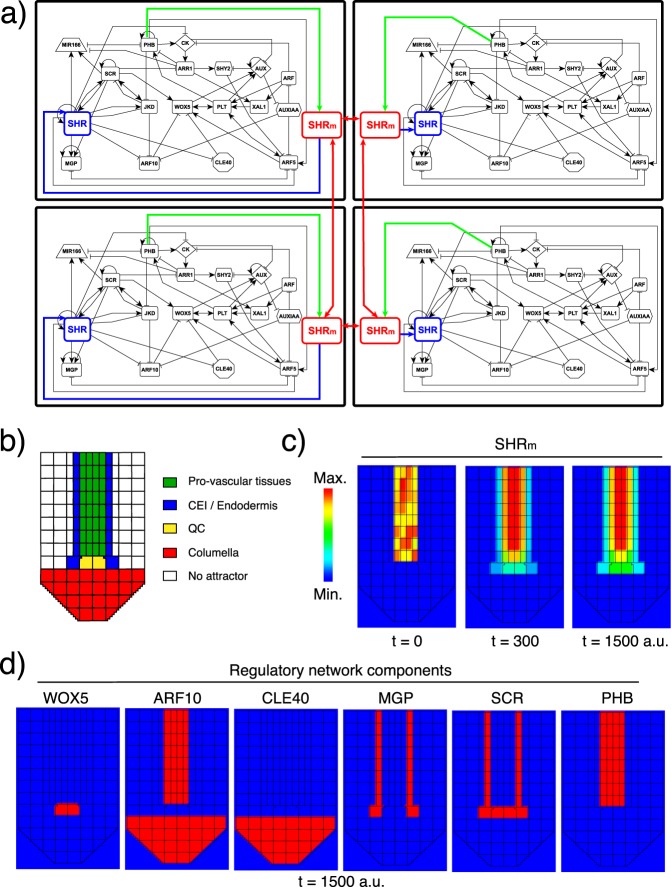
Multi-scale model of SHR intercellular transport in the root SCN. (**a**) Scheme of four cells of the multi-scale model, showing the links connecting the dynamics of their intracellular regulatory network through SHR_m_ intercellular movement. (**b**) Cellular configuration used as initial condition in the multi-scale model. The colours correspond to the attractor initialized in each cell. (**c**) Dynamics of SHR_m_ field formation: SHR_m_ is initially only present in the pro-vascular cells where its produced, while at t = 1500 a.u. it is also present in the layer adjacent to the pro-vascular cells, but no further. (**d**) Activity of six regulators of the regulatory network in the multicellular template. Time (t) is in arbitrary units (a.u.).

To understand the mechanism involved in creating variations in SHR levels in the progeny of a QC cell, and possibly in the production of columella initials, we next considered the constraints of *SHR*’s expression pattern and protein mobility in the root SCN. *SHR* is expressed in the pro-vascular cells of the root meristem, and its protein is transported to the QC cells, the CEI, and endodermal cells^55^. In this cell layer adjacent to the pro-vasculature, SHR forms protein complexes with JKD and SCR that localize to the cell nucleus, preventing SHR’s further intercellular movement^62,63^. These protein complexes also promote the expression of *SCR*, *JKD*, and other genes^54,62,64–66^, establishing a positive feedback loop. To study the coordinated role of SHR intercellular movement and the activity of the intracellular regulatory network in the dynamics of cell fate attainment in QC divisions, we developed a multi-scale model of the root SCN (Fig. 3). This multi-scale model simulates different cell types in the root SCN, how they exchange SHR through its regulated transport, and how they respond to changes in the intracellular levels of SHR. Importantly, each cell in the simulated cellularized domain has a regulatory network model that is coupled with the network of its neighbours through SHR transport, yielding a domain with a network of intracellular networks (Fig. 3a). The links that connect the regulatory network in each cell with the spatio-temporal information of SHR are explained below.

We used a static cellular configuration to model the cells of different cell types in the root SCN. The regulatory network model (Fig. 3a) of each cell was initialized into one of the following attractors: QC, CEI/Endodermis, central pro-vascular tissues, and columella initials (Fig. 3b). The state of the network in each cell was updated periodically throughout the simulation. We also included a continuous variable in each cell, SHR_m_ (the subscript m indicates that it models SHR movement), that can be exchanged between neighbouring cells periodically (Fig. 3a). These two cell properties, the intracellular regulatory network and the mobile variable SHR_m_, are linked such that the state of the network constrains the site of production of SHR_m_ while the intracellular levels of SHR_m_ feedbacks on the activity of SHR in the network (Fig. 3a). This means that SHR_m_ will be produced only in the pro-vascular cells, and that the activity level of SHR_m_ in a cell defines if it regulates its targets of the regulatory network or not. Finally, the intercellular transport of SHR_m_ was modelled considering the restrictive role of JKD and SCR, such that if they are active in the network of a cell, its transport will be much lower than if these regulators are inactive. Hence, we put forward a multi-scale model as schematized in Fig. 3a.

The multi-scale model initially recovered the activity of SHR_m_ exclusively in the pro-vascular cells as these are the only cells that can produce it (Fig. 3c). Then, SHR_m_ was also found in the adjacent layer to the pro-vascular tissues, composed of the QC, the CEI and the endodermal cells. This activity pattern of SHR_m_ did not change through the simulation (Fig. 3c) and corresponds to what has been described experimentally. There are three main parameters in the multi-scale model: SHR_m_ transport, SHR_m_ synthesis rate, and SHR_m_ activity threshold. We found that the distribution pattern of SHR_m_ we described above is robust to changes in these model parameters (Methods), and does not depend on a specific set. Indeed, many different parameter sets can reach this distribution (Supplementary Fig. 2). For the following simulations, we chose a parameter set that better describes the SHR’s observed behaviour in the root meristem and that complies with transport ratios described previously^67^. Importantly, the network’s attractors in the cells remain as initialized throughout this simulation (Fig. 3d); this result was dependent on the modelled dynamics of SHR_m_ intercellular movement (Supplementary Fig. 2). Thus, the multi-scale model—that considers the coordinated role of the dynamics of SHR_m_ movement and the regulatory network—accurately depicts the activity pattern of SHR as observed in the Arabidopsis root SCN.

Then, we put forward a multi-scale model of the constraints of the interaction of polarizing signals from the niche microenvironment (SHR) and the underlying regulatory network.

### SHR intercellular transport and the dynamics of the gene regulatory network guide the transition from QC to columella initials cell fate in the root SCN

**Figure 4:**
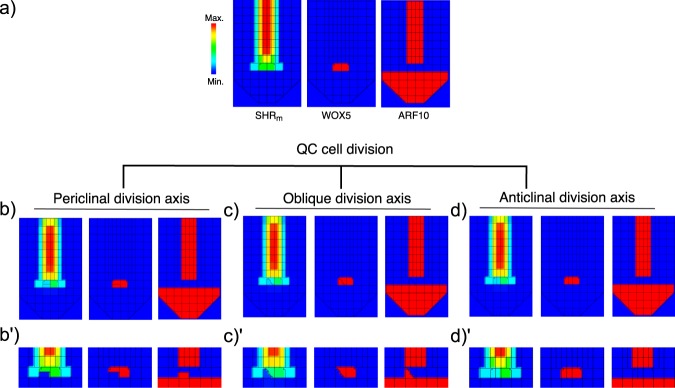
Simulation of a QC cell division in the multi-scale model. The activity pattern of SHR_m_ and the network regulators WOX5 and ARF10 are shown (**a**) before the division, (**b,c**, and **d)** immediately after the division takes place, (**b′,c′**, and **d′)** and some time after the division took place. (**b**) A periclinal division axis produces a shootward and a rootward daughter cell that gradually develop differences in SHR_m_ concentration and the states of their regulatory network: one cell remains at the QC attractor while the other shifts to the columella initials attractor. (**c**) The same pattern is observed for oblique divisions, where the division axis deviates from that of a periclinal or anticlinal pattern. (**d**) An anticlinal division axis produces two daughter cells that remain with high levels of SHR_m_ and in the QC attractor.

We used the multi-scale modelling platform presented in the previous section to simulate the division of a QC cell (Fig. 4). We assumed that upon a QC cell division, both daughter cells inherit the state of the intracellular regulatory network in the QC attractor (supported by time-lapse experiments^46^ and by the detection of equal levels of SHR_m_ in cell divisions of the adjacent CEI cells^55^). Then, we followed the fate of the QC’s progeny using the activity of the network’s regulators WOX5 and ARF10 as markers of QC cell and the columella initial cell states, respectively. The division axis of the QC cells was chosen at random in 100 simulations, and the results were classified as periclinal (25), anticlinal (31) and oblique (44) division axes.

We noticed that the simulations of a periclinal and an oblique division axis produced one rootward and one shootward daughter cell, that at the moment of the division, were identical (Fig. 4b,c). However, as the simulations ran, these cells developed differences in their intracellular levels of SHR_m_, and the rootward daughter cell transitioned to the columella initials attractor (Fig. 4b′). The dynamics recovered in the simulation of a QC periclinal division correlate with those reported experimentally^46^. In these experiments, the daughter cells initially maintain the activity of a QC marker (Fig. 4b) and eventually develop differences in its activity. The model shows that this asymmetric differentiation happens because of the differential proximity of the daughter cells to the pro-vascular cells. On the one hand, the shootward daughter cell, being in the immediate vicinity of the pro-vascular tissues, directly acquires and sequesters SHR_m_ maintaining its intracellular regulatory network in the QC attractor. On the other hand, the rootward daughter cell is one cell away from the pro-vascular tissues, and therefore, does not receive such large amounts of SHR_m_. In this latter case, the SHR_m_ levels inherited after the division gradually decay until the intracellular dynamic regulatory network of the rootward daughter cell transitions towards the columella initials cell-fate attractor. Importantly, the simulations suggest that the asymmetric QC divisions may not result from the divisions themselves, but rather from the interaction between the intracellular regulatory network and the cellular levels of SHR_m_.

In contrast to the above situations, the simulation of an anticlinal QC division axis produced two daughter cells that remained in the QC attractor (Fig. 4c). This symmetric division occurred because the daughter cells are equidistantly located from the pro-vascular tissues and thus received SHR_m_ from the primary source of the root SCN. The high levels of SHR_m_ in the daughter cells allowed them to remain in the QC attractor in all simulations of an anticlinal division axis.

Overall, the results of these simulations provide a system-level mechanistic explanation to why, in a periclinal QC cell division (the most common pattern in Arabidopsis root apices), one of the daughter cells differentiates into columella initials (the most common cell type^46^). Our multi-scale model simulations suggest that the same underlying systemic mechanism can yield both symmetric and asymmetric divisions, depending on the division axis of the QC cells, suggesting that QC cell divisions follow the population asymmetry model.

### Increasing the intercellular range of SHR activity causes a shift from asymmetric to symmetric QC periclinal divisions: Computational and experimental evidence

Our computational simulations led us to propose that SHR availability is a limiting factor in the decision to generate symmetric or asymmetric divisions in the QC (Fig. 4). We then hypothesized that increasing SHR’s activity range would have an impact on the fate of the daughter cells produced by QC periclinal divisions. There are several ways to increase SHR’s activity range, for example, if SHR protein has a higher mobility range^68^ or by expressing *SHR* in cells that typically do not express it^55^. We explored both possibilities using computational simulations and present the results here together with experimental data to support our theoretical proposals.

**Figure 5:**
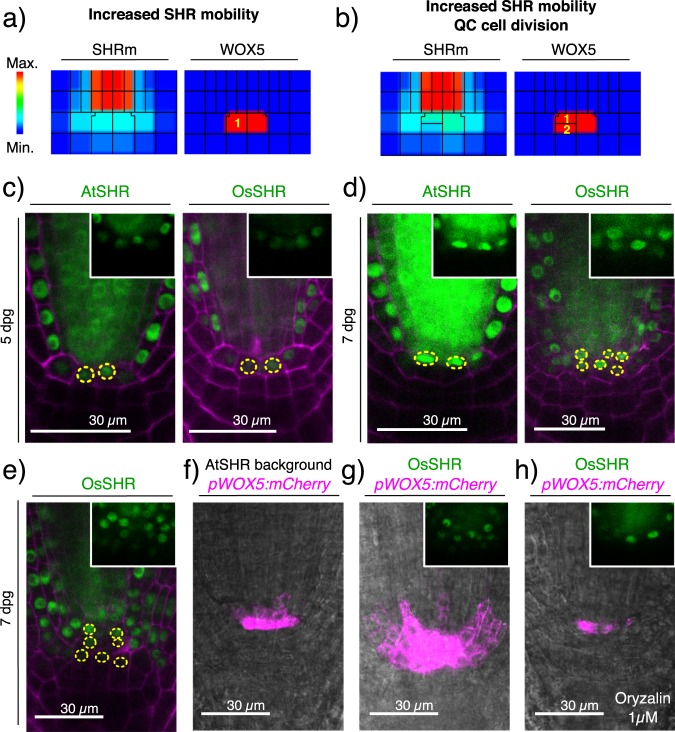
Analysis of SHR increased mobility range and its effect on the fate of the progeny of QC cell divisions. (**a**) The simulation of an increase in SHR_m_ transport rate causes an increased SHR_m_ activity domain, yet a single layer of QC cells is found below the pro-vascular cells (WOX5 as marker of the QC attractor). (**b**) The simulation of a periclinal division of a QC cell in this context results in the symmetric production of two cells with WOX5 activity. (**c**) Single layer of GFP nuclear activity in the QC position of 5dpg AtSHR and OsSHR seedlings. (**d**) 7dpg seedlings of AtSHR have a single layer of GFP nuclear activity, while most OsSHR seedlings have two or more (**e**). Analysis of *WOX5:mCherry* (magenta) in 7dpg WT and OsSHR seedlings: the expression of WOX5 is delimited to the QC cells in 7dpg WT seedlings (**f**), while its expression is expanded to several cell layers in 7dpg OsSHR seedlings (**g**). (**h**) 7dpg OsSHR seedlings treated with oryzalin (1 µM) for 6 hours have a single layer of nuclear GFP and WOX5 signal in the cells at the QC position. Overlay of GFP nuclear activity and propidium iodide counterstain (magenta) in (**c**), (**d**) and (**e**). All insets show the GFP signal from the respective SHR translational reporter.

The first way to increase the activity range of SHR is through increased protein mobility. We implemented this computationally by increasing the parameters related to SHR_m_ intercellular transport. In comparison to the WT simulation (Fig. 4a), the simulation of increased SHR_m_ transport resulted in higher SHR_m_ levels in several cell layers adjacent to the stele, which, in the context of the SCN, are the QC and columella initials cells (Fig. 5a). We noticed that despite the relatively higher levels of SHR_m_ past the stele, the *in silico* roots retained a single QC layer (one WOX5 + layer). This can be explained by the constraints of the regulatory network as SHR activity is not sufficient to cause an attractor transition from the columella initials towards the QC attractor (Supplementary Fig. 3c). This simulation then predicts that even if SHR levels are high in the columella initials, these will not become QC cells and there will be a single layer of QC cells. Then we simulated a QC periclinal division and found that, in this case, both daughter cells remain in the QC attractor (Fig. 5b). Therefore, a symmetric division took place. This simulation predicts that, in the context of increased SHR protein mobility, a QC periclinal division will yield two QC cells (Fig. 5b).

To assess these predictions experimentally, we used the *pSHR:OsSHR2:GFP* (OsSHR) translational reporter that expresses a rice orthologue of SHR under the endogenous Arabidopsis promoter^68^. It was previously shown that the OsSHR protein has more mobility than Arabidopsis’ AtSHR-GFP^68^, such that GFP signal was detected in up to 6 layers past the stele. This unique feature of OsSHR2 provided us with the opportunity to experimentally study the fate of the daughter cells produced by QC cell divisions in a condition of increased SHR transport. First, for comparison with our simulations, we aimed to establish a condition of infrequent and frequent QC cell divisions in OsSHR, and *pSHR:AtSHR:GFP* (AtSHR) as a control. A recent report found that QC cell divisions are more frequent in older plants^43^. Thus, we compared the frequency of QC periclinal cell division events in 5 and 7 dpg seedlings, and indeed we found that QC divisions were significantly more frequent in older seedlings in both lines (Supplementary Fig. 3). Having established conditions of infrequent and frequent QC cell divisions, we compared the number of QC cells in both lines. For this, we used the nuclear localization of GFP in the cells at the QC position as a marker. At 5 dpg, there was no significant difference in the number of cell layers with nuclear GFP between OsSHR and AtSHR as both lines exhibit a single layer (Fig. 5c). This is consistent with the computational simulations that show that even if SHR mobility increases, a single layer of QC cells will be found in the absence of cell divisions (Fig. 5a). In contrast, at 7 dpg the pattern between both lines was significantly different (P = 0.02; Supplementary Fig. 3); the OsSHR line had a higher frequency of multiple layers with nuclear GFP activity than the AtSHR line (Fig. 5d). Furthermore, some of the 7 dpg OsSHR seedlings had up to three layers of cells with nuclear GFP signal cells in the QC position (Fig. 5e), suggesting multiple rounds of QC periclinal divisions. This phenotype was never observed in AtSHR seedlings. The difference in the number of nuclear GFP layers in the QC position in both lines is consistent with the predictions from the simulations (Figs. 4b and 5b): in WT conditions, QC cell divisions will be asymmetric because not sufficient levels of SHR reach one of the daughter cells, whereas in the condition of increased SHR transport both daughter cells receive enough SHR to remain as QC cells.

To verify the identity of the additional nuclear GFP cell layers found in 7dpg OsSHR, we analysed the activity of *pWOX5:mCherry* in this line. *WOX5* is a gene that is expressed exclusively in the QC cells, and it is also a regulatory target of SHR^32^. Whereas WOX5 expression is normally demilited to a single layer of QC cells beneath the pro-vascular cells, even at 7dpg (Fig. 5f), we found multiple cell files with *WOX5* activity in OsSHR (Fig. 5g). This indicates that the additional cell layers have QC identity, and are likely the result of QC symmetric divisions. This result also shows that SHR is acting as a transcriptional regulator in the additional QC cell layers, as *WOX5* expression requires the activity of SHR^32^. Next, we aimed to show that the increased number of *WOX5* positive cells in OsSHR 7dpg seedlings is a consequence of the increased availability of SHR. To do this, we treated 7dpg OsSHR *pWOX5:mCherry* seedlings with the microtubule depolimerizing drug, oryzalin (1 µM for 6 hours as in^69^). Oryzalin has been shown to decrease SHR transport in the root meristem^69^. Strikingly, we found that the number of cell layers with GFP and *WOX5* signal was dramatically reduced in 7dpg OsSHR *pWOX5:mCherry* seedlings treated with oryzalin (Fig. 5h; n = 12 oryzalin and n = 24 DMSO treated seedlings, *Pearson’s Chi-square test with Yates’ continuity correction, P-value = 0.008665). These results clearly indicate that the multiple cell layers with QC identity observed in OsSHR are indeed a consequence of the increased intercellular transport of SHR; and thus, when reduced, the supernumerary layers disappear. Altogether, this experimental evidence supports that an increased range of SHR activity, via augmented protein mobility, can cause a shift from asymmetric to symmetric QC divisions, expanding the pool of undifferentiated stem cells (Fig. 1b). This had already predicted this by the multi-scale model simulations (Fig. 5), and the experiments we present here confirm it.

Next, we explored the effect of ectopically expressing SHR in the QC cells, as an alternative strategy to increase the range of activity of SHR. This was previously explored with the *pSCR::SHR* line that expresses *SHR* in the QC, CEI and endodermal cells^55^ (normally SHR is only expressed in the stele). The authors found activity of a QC marker in several cell layers between the stele and the root cap in the *pSCR::SHR* line^55^. To understand the mechanistic basis underlying this phenotype with multiple QC layers, we used the multi-scale computational model to simulate the *pSCR::SHR* line. To do this we included the ectopic production of SHR_m_ in the cells with the regulatory network in an attractor showing SCR activity (QC, CEI and endodermis). This simulation reached a distribution pattern with relatively high SHR_m_ levels in the two cell layers beneath the pro-vascular tissues, corresponding to the QC and the columella initial cells, while WOX5 was found active only in the cells in direct contact with the pro-vascular cells (Supplementary Fig. 3). Next, we simulated a periclinal division of a QC cell, finding that—contrary to what happens in WT—both daughter cells remained in the QC attractor. According to our simulations, this shift from asymmetric (WT) to symmetric cell fates (*pSCR::SHR*) happened because in the latter case the daughter cells produce SHR_m_ themselves and do not depend on the pro-vascular tissues as a source of SHR_m_ (Supplementary Fig. 3). Therefore, the daughter cells maintain high intracellular SHR_m_ levels and their networks remain indefinitely in the QC attractor. Linking this result with the reported phenotype of the *pSCR::SHR* line, we hypothesize that multiple cycles of QC periclinal divisions are behind the multiple QC layer phenotype reported by Nakajima and collaborators^55^. Thus, our computational simulations also provide a plausible systemic mechanistic explanation for the QC phenotype observed in the *pSCR::SHR* line.

These computational and experimental findings support our proposal that the feedback between the intracellular regulatory networks and the modulation of SHR’s intercellular transport, underlies cell fate decisions during the periclinal division of the QC cells in the root SCN.

### Non-equivalent QC cell state transitions in two experiments that affect the activity of QC-identity regulators

Next we aimed to assess the effects of reducing the range of activity of SHR. Recently it was shown that inhibiting SHR symplastic movement in the QC cells (with the *pWOX5:icalsm3m* line) results in decreased SHR signal, and a reduction in the expression of *SCR* and *WOX5* in these cells^70^. Intriguingly, these authors reported that the QC cells accumulated starch granules after the treatment, indicating that these cells differentiated into columella^70^. This phenotype is difficult to interpret, as not even *wox5* mutants have such drastic phenotypic alterations in QC cell identity^32^. We propose that the contrasting phenotypes of these two genetic perturbations (*pWOX5:icalsm3m* and *wox5*) are due to different alterations of the attractor landscape. To test this hypothesis and understand the system-level mechanistic basis underlying the phenotypes of these two genetic perturbations, we simulated both in our multi-scale model.

**Figure 6:**
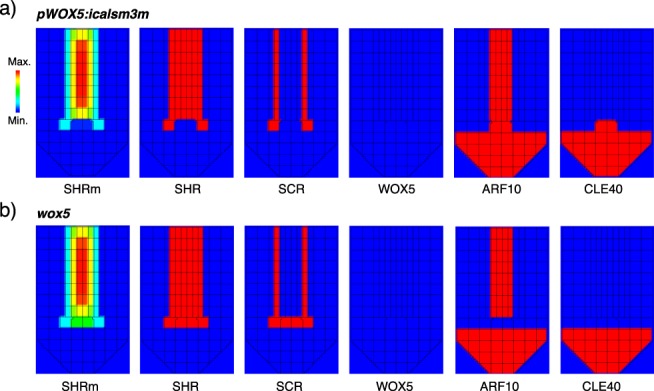
Simulation of two lines with contrasting QC cell identity defects. (**a**) Simulation of the *pWOX5:icalsm3m* inducible line. The inhibition of symplastic transport in the QC limits SHR_m_ movement and excludes it from these cells. The loss of SHR_m_ triggers a transition of the intracellular regulatory network from the QC to the columella attractor. (**b**) Simulation of the *wox5* mutant. In this mutant, the distribution of SHR_m_ is unaffected and the QC cells’ network is in a QC-like attractor in which the activity of various QC-related genes is maintained (see SHR and SCR).

The simulation of the *pWOX5:icalsm3m* line was implemented by not allowing SHR_m_ movement between the pro-vascular and the QC cells. We found that the levels of SHR_m_ gradually decreased in the QC cells, and eventually, these were so low that they were unable to activate SHR targets in their intracellular regulatory networks (Fig. 6a). This lack of activities caused the QC cells to become columella. The result of this simulation suggests that in the *pWOX5:icalsm3m* line the QC cells become columella cells because of how it limits SHR transport and how that feeds into the dynamics of the intracellular regulatory network. It also suggests that the minimal regulatory network model we used^53^ incorporates the key regulators to explain this intriguing phenotype. On the contrary, when we simulated the *wox5* mutant, we found that SHR_m_’s distribution was unaffected (Fig. 6b). The difference found between the WT and the *wox5* mutant was the state of the intracellular regulatory network of the QC cells, that in the latter attains a QC-like attractor that has activity of SHR and SCR, but not of WOX5 (Fig. 2c). In agreement with this result, in the *wox5* mutant, the QC cell identity is compromised but not completely lost, as several QC identity markers are still expressed in the QC position^32^. Although both the *pWOX5:icalsm3m* and the *wox5* simulations do not have activity of the QC-specific marker WOX5, these perturbations are not equivalent, particularly in the activity pattern of SHR (Fig. 6). Therefore, the simulation of these two genetic perturbations provides an explanation to their reported contrasting phenotypes (Fig. 5), revealing important differences in SHR spatio-temporal dynamics that explain the differences in the state of the QC cells. Such apparently paradoxical results may be explained by considering how spatial information (SHR) reshapes the attractor landscape of the root SCN regulatory network model towards different attractors.

These examples clearly illustrate the enormous explanatory potential inherent in systemic dynamical models of regulatory networks, that consider the concerted activity of different regulatory elements at different scales. Also, it further supports the critical role of SHR in the transition from the QC to columella cell state predicted by our computational analysis, but it does so in the context of complex regulatory networks coupled through SHR movement.

## Discussion

Multipotent SCs self-renew and generate different cell lineages upon division. However, the mechanisms underlying the cell-fate decisions during SC divisions and the resulting SCN cellular patterns are not entirely understood. The root SCN is a useful model system to uncover the systemic regulatory mechanisms underlying cell fate. In the root SCN, the QC cells divide asymmetrically, with a preference towards yielding columella initials cells^46^, but this biased cell-fate attainment pattern has not yet been explained. In the present study, we propose that this bias pattern can be explained by considering the constraints in the regulatory networks underlying cell fate decisions and signals from the root SCN microenvironment. To test this hypothesis, we used a dynamic gene regulatory network model that recovers attractors corresponding to the genetic activity configuration patterns of the cells of the root apical meristem, including those of the root SCN^53^. To explore the attractor landscape that emerges from the network’s interactions, we simulated perturbations of individual regulators to uncover the ones that can cause deterministic transitions among the system’s attractors^14,37,40^. Experimentally, this sort of analysis would require us to modulate the activity of each regulator in particular cell types of the root SCN and to monitor the cell-fate changes after each alteration, all of which is very time-consuming and challenging to achieve with the available tools. Alternatively, our computational analysis produced immediate hypotheses that can pose specific predictions and then inform future experiments, as the ones we present in this study. Although, many of the predictions of this analysis remain to be validated, the results we present here provide an overall system-level mechanistic framework to delimit the possible role of different network components in cell-fate transitions in the root SCN, and it thus constitute a valuable resource for the community.

The candidate regulators we identified through the decay-rate analysis (Fig. 2) can be interpreted as key regulators of differentiation, de-differentiation, and cell fate transition events at the root SCN and meristem. Importantly, the decay-rate analysis identifies the key regulators that could be targets of developmental, physical, chemical, and environmental cues to regulate the generation of certain initial cells in the root SCN. Regarding the transitions from the QC attractor to other fates, this analysis predicted regulators that can be important for the production of each of the initial cell-types by these reserve of multipotent SCs in the root SCN (Fig. 2c). Remarkably, we found that SHR is the only regulator whose downregulation can cause the transition from the QC to the attractor corresponding to the columella initial cells. The fact that this is the only regulator we found is noteworthy given that QC cells receive SHR, a mobile protein, from pro-vascular neighbouring cells, and other regulators of the network tightly control SHR’s movement. Therefore, SHR mobility could be informing QC cell-fate decisions within the niche.

To explore the role of SHR intercellular mobility in the asymmetric division of the QC cells, we developed a multi-scale model of the root SCN in which each simulated cell has an intracellular regulatory network linked to its neighbours’ network through the movement of SHR. This model is a tool in which we assessed the concerted role of SHR’s movement and the activity of the network in each cell, and with which we showed that the symmetry/asymmetry of QC cell divisions depends on the daughter cells’ locations relative to the source of SHR in the meristem (pro-vascular tissues). If there is a differential proximity, there will be a gradual decrease in SHR levels in the cell that is not in contact with the SHR source (rootward cell) and its intracellular regulatory network will stabilize in the columella initials attractor. Our decay-rate variation analysis already predicted this critical role of SHR in the transition from the QC to the columella initials attractor, and the multi-scale model complemented and further supported this prediction by considering the spatiotemporal dynamics of SHR intercellular transport in the root SCN. Importantly, our results support that, in the root SCN, QC divisions are uncoupled from cell differentiation. In this case, two identical cells are produced and the asymmetry in the fate of the progeny emerges from the joint dynamics of the intracellular regulatory network, the intercellular movement of SHR, and the stereotypical division axis.

To further support the role of SHR as a key regulator of cell-fate decisions in QC asymmetric divisions, we combined computational and experimental approaches to test how SHR activity range may alter cell-fate dynamics. We analysed cell-fate patterns in a transgenic line with increased SHR protein mobility^68^ and in a line with ectopic *SHR* expression^55^. In both cases, there was an increased number of cell layers with QC cell characteristics^68^ (Fig. 5 and Supplementary Fig. 3). Our computational simulations led us to propose that these phenotypes emerge due to QC periclinal cell divisions, in which both daughter cells remain as QC cells because of the increased availability of SHR (Fig. 5 and Supplementary Fig. 3). Indeed, we found that the multiple QC phenotype in the 7dpg OsSHR seedlings was reversed when we treated the plants with oryzalin (Fig. 5h), an inhibitor of SHR transport. We also explored the effect of decreasing the activity range of SHR. In this case, we found that QC cells differentiate into columella initials in the computational simulation (Fig. 6), which agrees with experimental observations^70^. Thus, both experiments and simulations that perturb SHR’s range of activity confirm the critical role of SHR in cell fate transitions from QC to columella initials.

Interestingly, the simulation of intermediate SHR levels in the regulatory network recovers an attractor with co-activity of QC and columella initials regulators (Fig. 2f). It has been reported that during the regeneration of the root meristem, the cells that eventually give rise to a new QC go through a phase where they have mixed QC-columella cell identity^61^. Remarkably, there is a correlation between the detection of this mixed QC-columella cell identity and the time it takes to restore SHR’s nuclear pattern in the QC position^61^. This correlation suggests that SHR could be the signal underlying this mixed cell fate, and that the feedback between SHR levels and the regulatory network reported here might be relevant not only for QC cell divisions but also in the context of root regeneration.

In line with experimental observations, the multi-scale model did not recover the production of initial cell types other than the columella initials^46^. Nevertheless, we predicted which candidate regulators’ downregulation could yield such transitions (Fig. 2c). The simulation of an anticlinal division axis of a QC cell produced two cells that remain QC cells (Fig. 4b), but a recent report showed that, in fact, an anticlinal QC division yields a QC and a CEI cell^48^. The decay-rate analysis we performed predicted that a decrease in the activity of AUX, PLT, or ARF5 could cause the transition from the QC to the CEI (Fig. 2c). It is possible that the growth of the daughter cells produced by the QC anticlinal division would cause the mechanical displacement of one of the cells out of the QC position, giving rise to auxin variations that could cause a transition of one of them to the CEI/Endodermis attractor. In order to recover such patterns, future improvements of the multi-scale model should incorporate the mechanics of cellular growth, detailed cell geometry, and the dynamics of polar auxin transport. Future model extensions should also address the role of cell geometry, mechanical forces^71,72^, auxin cellular concentrations^73^, and global cellular connectivity^74^ in the mechanisms that define the division axis of the QC cells or organizer cells in other SCN.

SCs are important for two critical processes in post-embryonic development: regeneration and differentiation. Through cell division, SCs regenerate themselves and also produce progeny that will differentiate and replenish functional tissues. SCs’ invariantly asymmetric divisions are usually assumed to be the basis for these cells’ long-term survival and the correct replacement of functional tissues. Nonetheless, recent lineage-tracing analyses have shown that asymmetry can also be achieved at the population level if SC divisions are uncoupled from cell differentiation^4–10^. So far, this population asymmetry model has only been described in animal SCNs. Here, through a combined computational and experimental approach, we provide evidence of QC cells following a population-level asymmetry model in the root SCN. In this scenario, a QC division produces two identical QC cells and afterward, key regulators (SHR) signal the progeny’s regulatory networks, in some cases yielding the cell-fate transition of one of the daughter cells. Indeed, symmetric and asymmetric QC cell divisions were recovered by our simulations (Figs. 4 and 5) by altering the range of activity of SHR and its availability to the QC daughter cells. Therefore, we propose that symmetric and asymmetric divisions can emerge from the same regulatory mechanisms at play in different spatial domains. This proposal implies that SCs do not follow a pre-established lineage program, but rather that their cell-fate decisions emerge from the interplay between their pre-existing cell state and the conditions of the microenvironment. The apparent regularity of the observed patterns depends upon a robust mechanism, in which constraints in the direction of the division axis are also involved. We recognize that other regulators may play a role as well, and do not claim that the mechanism described here is complete.

Stereotypical patterns of cell-fate attainment have been observed in many SCNs and show a strong correlation between the SC division axis, the position of the progeny after a division, and their fate^46,75–78^. Interestingly, these patterns suffer alterations upon deviations of the division axis in several systems^77,79^, suggesting that the division axis influences how daughter cells will be affected by critical positional signals. The theoretical platform we present here provides the first system-level mechanism to study the contribution of the division axis and feedback between intra- and intercellular dynamics during fate decisions at a SCN. Our approach may be useful to decipher the complex mechanisms guiding SC cell fate dynamics in other SCNs. Indeed, systemic mechanisms similar to the one we presented here may be involved in both cell-fate attainment and morphogenetic patterning in other plant and animal SCNs. Thus, both the systemic mechanism and the modelling framework proposed in this study may become useful to further understand the mechanisms involved in plant and animal SCN cell patterning and replacement dynamics.

## Methods

### Regulatory network model

To study the mechanisms underlying cell fate transitions we used an extended and updated version of a Boolean network model that we previously proposed^53^. This Boolean network integrates the role of key transcription factors, hormones, peptides, and other regulators of cell fate in the root meristem. Through an extensive literature review, the regulatory interactions between all the elements were identified and abstracted as a network of interacting elements. The activity of each element of the network is modeled as a Boolean variable, such that it can be active (1) or inactive (0). Logical rules, one per each element of the network, are proposed; these formalize the experimental evidence (with the logical operators AND, OR, and NOT) and takes as input the state of its regulators. The logical rules take the general form:

1 $${x}_{i}(t+1)={F}_{i}({x}_{1}(t),\ldots ,{x}_{k}(t))$$

where *x*_*i*_ (*t* + 1) is the state of node *x*_*i*_ at time *t* + 1 and *x*_1_(*t*),…, *x*_*k*_(*t*) are the states of its regulators a previous timestep. By applying the logical rules iteratively we can update the state of the network from all possible initial conditions (2^number of nodes), until eventually the system reaches activity configurations that do not change anymore and are thus self-sustained. These are known as attractors and emerge due to the constraints considered in the model (namely, the logical rules that are based on experimental evidence). The attractors at which the system converges can be interpreted as the different cell types or fates of the biological system under study. In the case of the root SCN, the attractors recovered correspond to different cells of the niche including the QC, CEI, central and peripheral pro-vascular initials, and columella initials.

The Boolean network can be further extended to a continuous model in which the state of the nodes is no longer limited to 0 and 1, but can have any value between these range^80^. In the continuous version of the model, the activity of each regulator is described by an ordinary differential equation (ODE) with the form:

2 $$\frac{d{x}_{i}}{dt}=\frac{-{e}^{0.5h}+{e}^{-h{w}_{i}}}{(1-{e}^{0.5h})(1+{e}^{-h({w}_{i}-0.5)})}-{\gamma }_{i}{x}_{i}\,$$

The first term in (2) describes the production of *x*_*i*_ with a sigmoidal function, and the second term describes its linear decay. The parameter *h* determines the strength of the interactions and controls if the activation curve of a node resembles a step function, a logistic function, or a straight line; *γ* is the decay rate. By default, the *h* and *γ* parameters are the same for all the elements of the network. Nevertheless, we show in Supplementary information D that the results reported in this paper do not depend on the particular value of *h* used. The *w*_*i*_ is the continuous form of the logical function of node *i* using fuzzy logic as in^80^. Briefly, the logical operators OR, AND and NOT are substituted by a maximum function, minimum function, or a subtraction, respectively, as shown in the following examples:

$$\begin{array}{rrr}\underline{{\bf{Logical}}\,{\bf{function}}} &  & \underline{{\boldsymbol{w}}\,{\bf{function}}}\\ {{\rm{x}}}_{{\rm{i}}}({\rm{t}}){\bf{O}}{\bf{R}}\,{{\rm{x}}}_{{\rm{j}}}({\rm{t}}) & \to  & {\bf{M}}{\bf{A}}{\bf{X}}({{\rm{x}}}_{{\rm{i}}}({\rm{t}}),\,{{\rm{x}}}_{{\rm{j}}}({\rm{t}}))\\ {{\rm{x}}}_{{\rm{i}}}({\rm{t}}){\bf{A}}{\bf{N}}{\bf{D}}\,{{\rm{x}}}_{{\rm{j}}}({\rm{t}}) & \to  & {\bf{M}}{\bf{I}}{\bf{N}}({{\rm{x}}}_{{\rm{i}}}({\rm{t}}),\,{{\rm{x}}}_{{\rm{j}}}({\rm{t}}))\\ {\bf{N}}{\bf{O}}{\bf{T}}\,{{\rm{x}}}_{{\rm{i}}}({\rm{t}}) & \to  & {\bf{1}}-{{\rm{x}}}_{{\rm{i}}}({\rm{t}})\end{array}$$

The resulting *w*_*i*_ is then integrated into (2). For a more detailed description on Boolean models to model gene regulatory networks we recommend^36^, and for more details on the root apical meristem regulatory network we recommend^53^; the latter includes all the details of how the Boolean network model we used here was integrated from experimental data.

### Decay-rate variation analysis of the continuous regulatory network model of the root SCN

To find the regulators (nodes) whose alterations are sufficient to cause transitions in the model’s attractors, we performed a decay-rate variation analysis^39^. For this analysis we used the continuous version of the dynamic regulatory network model underlying cell-fate decisions in the root SCN^53^. First, we used as initial condition the activity configuration of one of the six attractors of the model. Then, we gradually increased the decay rate, γ^*^, of one of the active regulators in that attractor, solved the system until the derivatives became smaller than a threshold^81^ (meaning it reached a steady state), and added the value of all the regulators’ activity (i.e. $\mathop{\sum }\limits_{i=1}^{X}{x}_{i}$). This sum was plotted for each decay rate analysed for each initial attractor (Supplementary Tables C), and allowed us to visually asses if the increase in γ^*^ caused a sudden change in the network’s behaviour. This suggest that an attractor transition occurred (even when the initial and final attractor yield the same sum, a noticeable jump is seen) and shows the particular value of *γ* at which this transition may have happened.

We then analysed the activity configuration at which the system did not change anymore (final state). To associate a particular cell state to a final state, we compared the activity of each node to that of the attractors recovered by the intact model (Supplementary Tables S3). In some cases the final state was identical to one of the attractors and the association was straightforward. In other cases, the activity of one or more nodes did not match the original attractors; in such cases, as long as the cell-fate regulators had the correct activity patterns, we associated the final state to that attractor. These latter cases are clearly indicated in Supplementary Tables C and D (with bold lettering and asterisks). The decay-rate analysis was performed in the R programming environment with a code that systematically changes the decay rate of every active node for every attractor of the system, and retrieves the output plots (Supplementary Tables C).

### Multi-scale model of the SHR transport and intracellular regulatory network in the root SCN

We used the software CompuCell3D^82^ to propose a multi-scale model of the root SCN. CompuCell3D is a lattice-based computational framework that models individual cells as a collection of positions in a grid. In our simulations, the position of each cell is fixed. Cells act as cellular automata that can communicate with its neighbors according to a series of interaction rules. As an initial condition, we used a cellular configuration resembling the cell distribution in the Arabidopsis root SCN (Fig. 3b); here volumes are merely illustrative. Each of the cells has two important features: A Boolean model and an SHR_m_ variable. The Boolean model was initialized in the attractor corresponding to the cell type observed in the position of the QC, the Endodermis, the pro-vascular and columella cells. The network we used does not describe the activity configuration of the epidermis or the cortex cells of the root meristem^53^ and therefore, the network dynamics were not modelled in these cells. The SHR_m_ continuous variable models SHR transport between neighbouring cells. The Boolean model and the SHR_m_ are connected according to the following rules:

3 $${{\rm{SHR}}}_{{\rm{m}}}=\{\begin{array}{ll}{\rm{if}}\,{{\rm{PHB}}}_{{\rm{GHRN}}}=1 & {{\rm{synthesis}}}_{{{\rm{SHR}}}_{{\rm{m}}}}-{{\rm{\delta }}}_{{{\rm{SHR}}}_{{\rm{m}}}}{{\rm{SHR}}}_{{\rm{m}}}\\ {\rm{else}} & \,-\,{{\rm{\delta }}}_{{{\rm{SHR}}}_{{\rm{m}}}}{{\rm{SHR}}}_{{\rm{m}}}\end{array}$$

4 $${{\rm{SHR}}}_{{\rm{GHRN}}}=\{\begin{array}{ll}{\rm{if}}\,{{\rm{SHR}}}_{{\rm{m}}} > {\rm{threshold}} & {{\rm{SHR}}}_{{\rm{GHRN}}}=1\\ {\rm{else}} & {{\rm{SHR}}}_{{\rm{GHRN}}}=0\end{array}$$

5 $${{\rm{D}}}_{{{\rm{SHR}}}_{{\rm{m}}}}\,\{\begin{array}{ll}{\rm{if}}\,{{\rm{SCR}}}_{{\rm{GHRN}}}={{\rm{JKD}}}_{{\rm{GHRN}}}=1 & {{\rm{D}}}_{{{\rm{SHR}}}_{{\rm{m}},1}}={\rm{d}}\ast (0.5)\\ {\rm{else}} & {{\rm{D}}}_{{{\rm{SHR}}}_{{\rm{m}},0}}={\rm{d}}\end{array}$$

The first rule (3) confines SHR_m_ production to the pro-vascular cells^54^. The simulation evaluates, in each cell, if PHB is active in the regulatory network; if true, there will be SHR_m_ production and linear decay and if not, SHR_m_ will only decay linearly. The second rule (4) establishes an activity threshold that must be surpassed by the cellular level of SHR_m_ to activate SHR in the intracellular regulatory network, so that the transport dynamics feedback into the regulatory network. The third rule (5) models that the intercellular transport rate of SHR_m_ will depend on the state of the intracellular regulatory network, such that if SCR and JKD are active, the rate will be lower than if they are inactive. Recently, high-resolution microscopy imaging estimated that the diffusion of SHR from the endodermal cells is ~52% of the diffusion from the pro-vascular to the endodermal cells^67^. This ratio was incorporated in rule (4). Importantly, the multi-scale model explicitly models the role of JKD and SCR in the regulation of SHR movement. Therefore, we did not consider this regulation in the regulatory network (compare network topologies in Figs. 1b and 3a).

A discretized Laplacian was used to model the intercellular movement of SHR_m_. This operator takes into account that the amount of SHR_m_ a cell receives from neighbouring cells (N) depends on their cell-type (4) (${{\rm{D}}}_{{{\rm{SHR}}}_{{\rm{m}},0}}$ or ${{\rm{D}}}_{{{\rm{SHR}}}_{{\rm{m}},1}}$) and that the amount of SHR_m_ sent to the neighbouring cells depends on its own cell type (*D*_*own*_):

6 $${{\rm{SHR}}}_{{{\rm{m}}}_{{\rm{i}},{\rm{j}}}}(t+1)={{\rm{SHR}}}_{{{\rm{m}}}_{{\rm{i}},{\rm{j}}}}(t)+{{\rm{D}}}_{{{\rm{SHR}}}_{{\rm{m}},1}}\mathop{\sum }\limits_{n}^{{N}^{\ast }}{{\rm{SHR}}}_{{{\rm{m}}}_{{\rm{n}}}}(t)+{{\rm{D}}}_{{{\rm{SHR}}}_{{\rm{m}},0}}\mathop{\sum }\limits_{n}^{{N}^{\ast \ast }}{{\rm{SHR}}}_{{{\rm{m}}}_{{\rm{n}}}}(t)-4\,{{\rm{D}}}_{{\rm{own}}}{{\rm{SHR}}}_{{{\rm{m}}}_{{\rm{i}},{\rm{j}}}}(t)$$

In (6), the symbol N^*^ is used to indicate the neighbours that have SCR and JKD activity in their intracellular regulatory network while N^**^ is used to indicate the neighbours that do not. *D*_*own*_ is the intercellular transport rate depending on the cell type of the cell being updated (can be either ${{\rm{D}}}_{{{\rm{SHR}}}_{{\rm{m}},0}}$ or ${{\rm{D}}}_{{{\rm{SHR}}}_{{\rm{m}},1}}$). The intercellular transport rates are defined by rule (4).

A limitation of the modelling framework we used for this multi-scale analysis is that it does not consider the shape of the cells nor the cell growth that occurs after the QC cell division; both can potentially alter the number of neighbors of the daughter cells and their levels of SHR. To test the effect of diffent cell alignments in the differentiation dynamics upon QC divisions we used three cellular configurations in which the daughter cells have different sizes and neighbours after the division (Supplementary Fig. 4). We found that the same differentiation patterns reported in the main text were recovered by these different initial cell configurations, showing that the patterning dynamics described are robust to changes in the number of cell neighbours and the alignment of the daughter cells. This is because the source of SHR is the main constraint underlying the differentiation dynamics described in this study.

### Parameter analysis of SHR_m_ transport in the multi-scale model

We performed a parameter analysis to test the robustness of the SHR_m_ distribution pattern to the quantitative parameters of the multi-scale model (SHR_m_ transport rates, SHR_m_ synthesis and SHR_m_ activity threshold). First, we varied the SHR_m_ transport rates, and then we varied the SHR_m_ synthesis and SHR_m_ activity threshold. In both cases, we analyzed 3,000 parameter sets and analysed the number of SHR_m_ + cell layers in the QC position (the cells below the pro-vascular cells with SHR_m_ levels higher than the SHR_m_ threshold).

In the first case, the SHR_m_ intercellular transport rates were varied within the range: 0.0001–0.01 a.u. (Supplementary Fig. 2). We chose a rate for the cells with SCR/JKD activity (QC, CEI, and Endodermis cells) and a different rate for the cells with no activity of these regulators (which we will refer as stele cells hereafter). We found zero SHR_m_ + cell layers when the transport from the stele cells was too low, a single SHR_m_ + cell layer when the transport from the QC/CEI/Endodermis cells was lower than the transport from the stele (as described for Arabidopsis roots^67^), and two SHR_m_ + cell layers when both transport rates were high. Interestingly, we also found the one SHR_m_ + cell layer pattern in a parameter subdomain where the transport rate from the QC/CEI/Endodermis cells was higher than the transport from the stele cells, but only for very low values of this second rate. This pattern is observed because, even though the rate from the QC/CEI/Endodermis cells is high and could potentially transport SHR_m_ to the following tissue layers, these cells do not receive high amounts of SHR_m_ in the first place (Supplementary Fig. 2). Recently, high-resolution microscopy was used to estimate the diffusion rate of SHR from the stele and the endodermis^67^. This study^67^ found the transport from the endodermis to be 52% lower than the transport from the stele. To explore if this ratio between both rates could be related to the single SHR_m_+ cell layer distribution observed in Arabidopsis, we identified the simulations that have such proportion (52% ± 2, yellow asterisks in Supplementary Fig. 2). As can be observed in Supplementary Fig. 2, the simulations with transport rates that satisfy this ratio could have either a single or two SHR_m_ + cell layers in the QC position. Thus, the ratio between both transport rates does not guarantee the existence of a single SHR_m_+ cell layer pattern in the simulation platform. Instead, the pattern of SHR_m_ + cells emerges from the coupled SHR_m_ transport dynamics in cooperation with the rest of the parameters and dynamics considered in the multi-scale model.

Next, we varied both the rate of SHR_m_ synthesis (values within the range 50–5,000 a.u.) and the SHR_m_ activity threshold (1,000–100,000 a.u.) and evaluated the number of SHR_m_+ cell layers. In this case, we found a more linear pattern where the number of SHR_m_+ cell layers increased as the SHR_m_ synthesis rate increased, and the number of layers decreased as the SHR_m_ threshold increased (Supplementary Fig. 2).

### Plant growth conditions and microscopy

The transgenic lines used in this study, *pSHR:AtSHR:GFP* (AtSHR throughout the text)^55^, *pSHR:OsSHR2:GFP* (OsSHR)^68^ and *WOX5:mCherry*, are in Arabidopsis’ Col-0 genetic background and were kindly shared by Joseph Dubrovsky, Kimberly Gallagher, and Ken Birnbaum, respectively. Seeds were sterilized in a 1 ml of 20% sodium hypochlorite and 0.02% Tween 20, stratified for two days in a 4°C dark room, and plated on a growth medium with 0.2X Murashige and Skoog (MS) salts, 1% Sucrose, 1% agar, and MES (pH 5.6). Seedlings were grown vertically at 22 °C in climate chambers in long day conditions (16 hours of light followed by eight hours of dark) for five and seven days. For confocal microscopy, roots were mounted in propidium iodide (PI, 50 µg/ml in MS 0.2X) and visualized using a Nikon Eclipse Ti-E confocal microscope. The images presented in this article correctly represent the original data.

### Oryzalin treatment

Following the conditions reported previously^69^, we transferred 7dpg seedlings to plates containing MS 0.2% agar with oryzalin at 1 µM in DMSO (dimethylsulfoxide) and observed in the confocal microscope 6 hours later. Controls were transferred to plates with the same amount of DMSO.

## Supplementary information

Supplementary Data.
