# Supplementary material for: A system-level mechanistic explanation for asymmetric stem cell fates: Arabidopsis thaliana root niche as a study system
Source: Sci Rep. 2020 Feb 26;10:3525. doi: 10.1038/s41598-020-60251-8 (PMC7044435; doi:10.1038/s41598-020-60251-8)
Supplement: Supplementary file 1 — Supplementary Data. [file 41598_2020_60251_MOESM1_ESM.pdf]

<u>Supplementary information</u>

A system-level mechanistic explanation for asymmetric stem cell fates: *Arabidopsis thaliana* root niche as a study system

Mónica L García-Gómez, Diego Ornelas-Ayala, Adriana Garay-Arroyo, Berenice García-Ponce, María de la Paz Sánchez and Elena R Álvarez-Buylla

Table of contents:

A. Supplementary Figures
Supplementary Figure 1
Supplementary Figure 2
Supplementary Figure 3
Supplementary Figure 4

B. Supplementary Methods. The updated regulatory network model of the *Arabidopsis thaliana* root apical meristem.
Supplementary Table S1 - Interactions included in the updated regulatory network model
Supplementary Table S2 – Logical rules
Supplementary Table S3 - Attractors recovered by the updated regulatory network model

C. Supplementary Tables: Decay-rate variation analysis
Supplementary Table S4 - Initial state: QC
Supplementary Table S5 - Initial state: CEI/Endodermis
Supplementary Table S6 - Initial state: Peripheral pro-vascular
Supplementary Table S7 - Initial state: Central Pro-vascular
Supplementary Table S8 - Initial state: Columella initials
Supplementary Table S9 - Initial state: Transition domain

D. Supplementary Tables: Parameter *h* variation
Supplementary Table S10 - Initial state: QC
Supplementary Table S11 - Initial state: CEI/Endodermis
Supplementary Table S12 - Initial state: Peripheral pro-vascular
Supplementary Table S13 - Initial state: Central Pro-vascular
Supplementary Table S14 - Initial state: Columella initials
Supplementary Table S15 - Initial state: Transition domain

E. References

A. Supplementary Figures

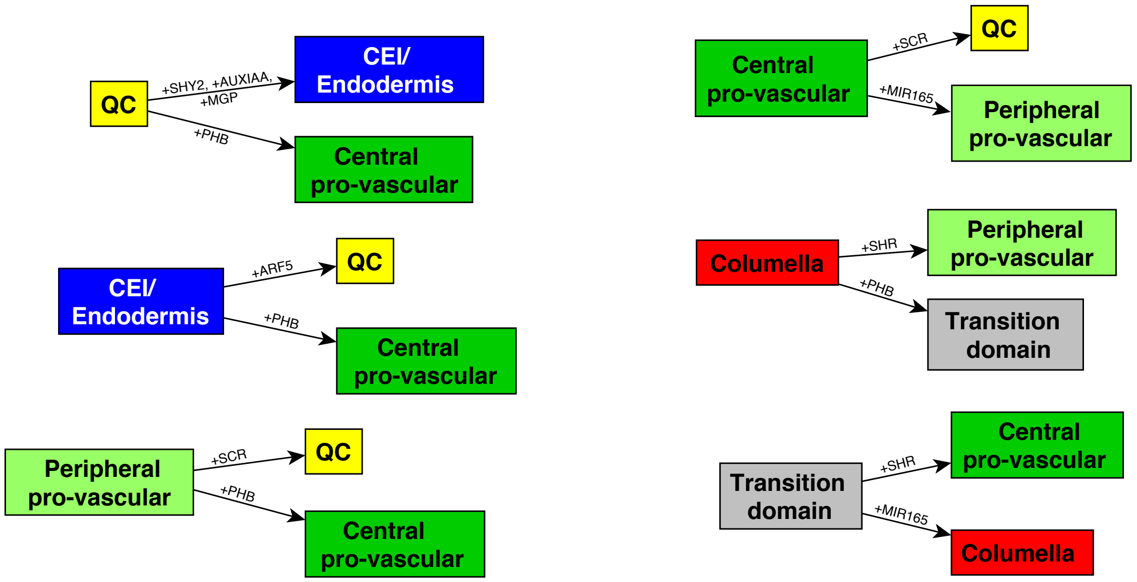

Supplementary Figure 1- Attractor transitions caused by the upregulation (+) of individual regulators that are normally inactive in the initial attractor are shown at the edges of each transition.

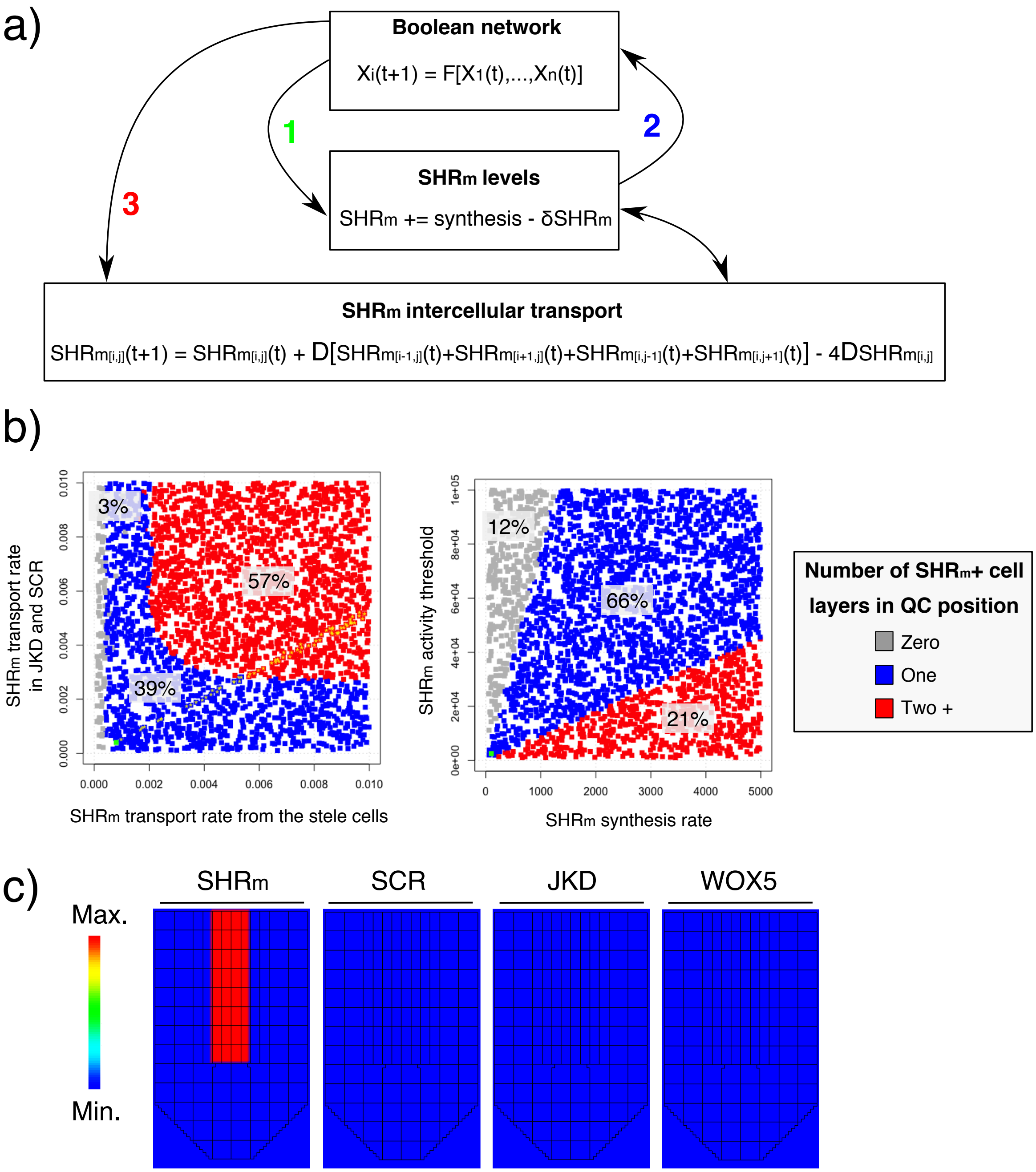

Supplementary Figure 2- (**a**) Diagram showing the coupling of the intracellular regulatory network and the mobile variable SHR_m. The state of the network defines the production site of the mobile variable SHR_m in the pro-vascular cells (1, blue), SHR in the network is activated if a SHR_m activity threshold is surpassed (2, green), and the transport rate of SHR_m is regulated by the state of the network (3). (**b**) Analysis of SHR_m quantitative parameters and their effect on the number of SHR_m+ cell layers (i.e., cells that surpass the SHR_m activity threshold) in the QC position. On the left we show the results of variations in SHR_m transport rate (for SCR/JKD positive cells and stele cells), and on the right the results of variations in

SHR_m synthesis rate and activity threshold. In each case, we explored 3,000 parameter sets taken at random from uniform distributions and plotted in gray, blue, or red the number of SHR_m+ cell layers for each analyzed set. (**c**) A simulation without SHR_m mobility recovers a state in which the continuous variable SHR_m is found only in the pro-vascular cells, and no activity of endodermal and QC markers was recovered.

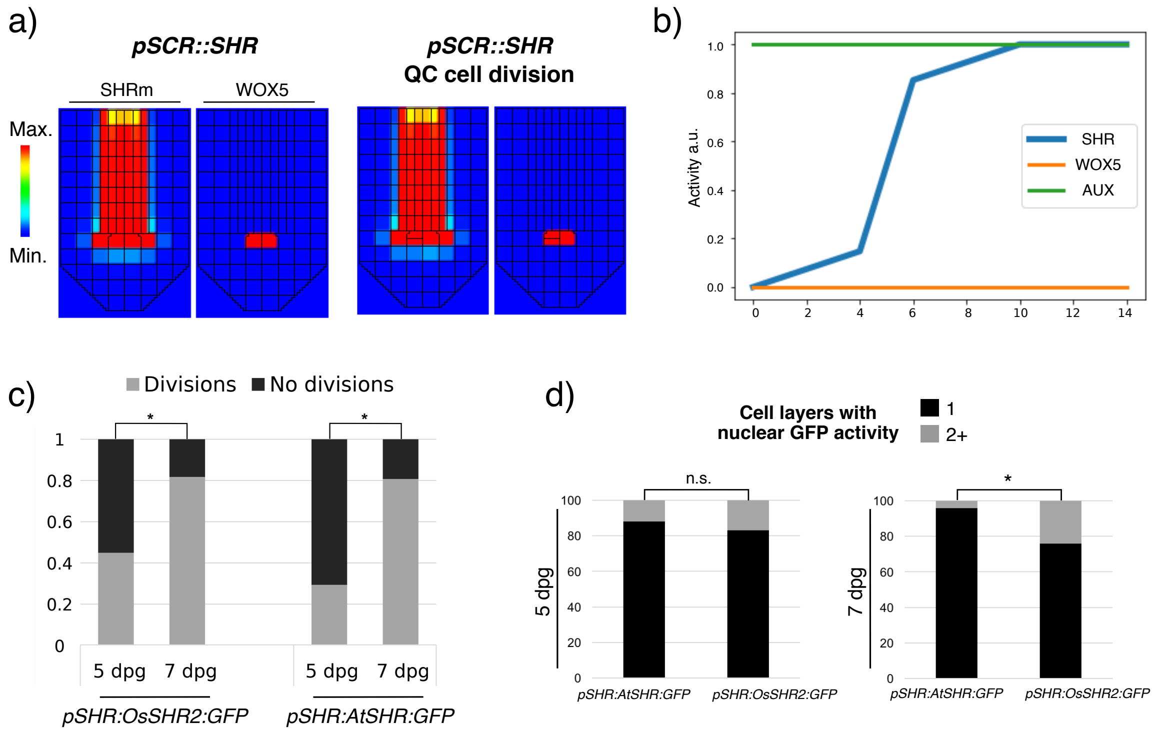

Supplementary Figure 3- (**a**) Simulation of the *pSCR::SHR* transgenic line: a single layer of QC cells below the pro-vascular cells was recovered, while the simulation of a QC periclinal division yields two QC cells. (**b**) The ectopic activation of SHR (blue) from the columella initials attractor does not activate WOX5 (orange), and therefore a transition to the QC attractor does not occur. (**c**) The frequency of morphologically identifiable QC periclinal divisions is significantly higher in 7 dpg plants than 5 dpg seedlings for the *pSHR:OsSHR2:GFP* and *pSHR:AtSHR:GFP* (*Pearson's Chi-square test with Yates' continuity correction, P-value= 0.0004201 and 0.0006645, respectively). (**d**) Analysis of the frequency of GFP nuclear signal in 5 dpg and 7 dpg *pSHR:AtSHR:GFP* and *pSHR:OsSHR2:GFP* lines. At 5 dpg, most plants analysed have a single layer of GFP nuclear signal; non-significant differences were found between both lines (n.s.) (n=29 for pSHR:OsSHR2:GFP and n=17 for *pSHR:AtSHR:GFP*). At 7 dpg, the frequency of seedlings with multiple layers of GFP nuclear signal in the QC position is significantly higher in *pSHR:OsSHR2:GFP* than in *pSHR:AtSHR:GFP* (n=79 for *pSHR:OsSHR2:GFP* and n=26 for *pSHR:AtSHR:GFP*, *Fisher's exact test, P-value=0.0223).

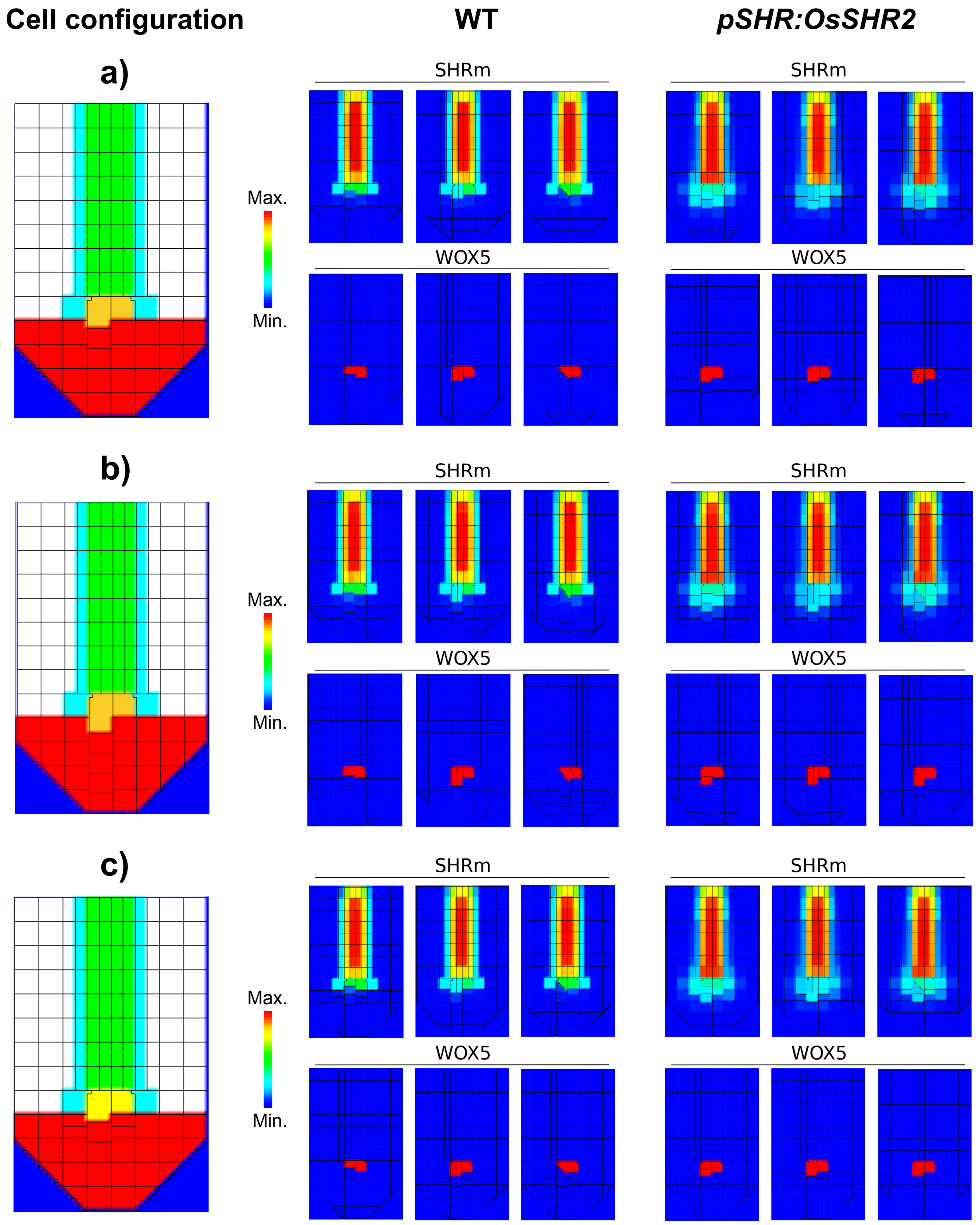

Supplementary Figure 4- Simulation of QC cell divisions using three different initial QC cell (**a**,**b**) and neighbouring columella initial (**b**) configurations. For each configuration we simulated periclinal, anticlinal and oblique QC division axes, in WT and increased SHR transport conditions (*pSHR:OsSHR2*). We show in each case the SHRm distribution (above) and the activity of WOX5 (below).

B. The updated regulatory network model of the *Arabidopsis thaliana* root apical meristem

We updated the Boolean model of the genetic-hormonal regulatory network (Figure 1B; García-Gómez et al., 2017) to consider the role of the MADS-box transcription factor XAANTAL1 (XAL1), the PLETHORA (PLT) transcription factors, as well as additional regulatory interactions among the pre-existing components of the network, according to recent experimental data (Table S1). The genes included in the model are: WUSCHEL-RELATED HOMEOBOX5 (WOX5), AUXIN RESPONSE FACTOR (ARFr) as a representative of all ARFs, AUXIN RESPONSE FACTOR5 (ARF5), AUXIN RESPONSE FACTOR10 (ARF10), XAL1, PLT, PHABULOSA (PHB), ARABIDOPSIS RESPONSE REGULATOR1 (ARR1), SHORT HYPOCOTYL2 (SHY2), AUX/IAA transcriptional regulator (AUXIAA), microRNA 165/6 (MIR166), MAGPIE (MGP), SCARECROW (SCR), JACKDAW (JKD), SHORTROOT (SHR), CLAVATA40 (CLE40); and the two hormones are: Auxin (AUX) and Cytokinin (CK).

Interaction	Experimental evidence
WOX5 -> PLT	An epistatic genetic interaction has been described between *wox5* and *plt1 plt2* mutants (Ding and Friml, 2010), suggesting that these PLT transcription factors act downstream of WOX5. Additionally, the expression of the transcriptional reporter *PLT1::ERCFP* is reduced in the *wox5* mutant background, this is very clear in the QC cells (Ding and Friml, 2010). Moreover, PLT1 expression is increased after the induction of WOX5 expression in a *p35S::WOX5:GR* line (Ding and Friml, 2010).
ARF5 -> PLT	*In situ* analysis has shown that the expression of PLT1 and PLT2 is greatly reduced in *mp* mutants in embryonic and post-embryonic root development (Aida et al., 2004).
ARF -> PLT	The expression of PLT1 is further reduced in the embryos of the double mutant *mp nph4* (Aida et al., 2004), indicating a redundancy in the action of the auxin signaling pathway.
XAL1 -> PLT	The transcriptional reporter *PLT1::GUS* is reduced in the *xal1-2* mutant, particularly in the QC and the initial cells (García-Cruz et al., 2016).
ARF -> XAL1	XAL1 expression is induced in roots exogenously treated with auxins as indicated by GUS activity in the *XAL1::GUS* transgenic line (Tapia-López et al., 2008). *In situ* expression analysis indicated that XAL1 is expressed in the columella and the lateral root cap, and has a punctuated expression pattern in all the cells of the root meristem (Burgeff et al., 2002). We assumed that this regulation is mediated by the auxin signaling pathway.
PLT -> AUX	The induction of PLT2 expression coupled with a transcriptomic analysis of the QC cells uncovered genes involved in auxin transport and metabolism among the genes with induced expression (Santuari et al., 2016). PLT2 binds directly to the promoter of YUC3 and YUC8, and accordingly, the *plt1 plt2* mutant has reduced auxin levels in root tips (Santuari et al., 2016). Moreover, induction of the *p35S:PLT2-GR* lines have increased auxin levels (Santuari et al., 2016).
PLT -> ARF5	PLT2 binds the promoter of *ARF5*, whose expression is induced upon DEX treatment of *35S:PLT2-GR* lines (Santuari et al., 2016).

PHB -> ARF5	*ARF5* expression is reduced in roots with reduced PHB activity (Baima et al., 2014; Müller et al., 2015). PHB binds to the promoter of *ARF5,* and induces its expression in DEX-treated *35S:PHB-GR* lines (Müller et al., 2015). The expression of *ARF5* is only slightly reduced in roots with reduced PHB activity, thus it is likely that its expression depends on other factors as well (Müller et al., 2015).
PLT -> PHB	PLT2 directly regulates the expression of *PHB* in the root meristem (Santuari et al., 2016).
ARR1 -| PHB	Cytokinin exogenous treatment reduces the expression of PHB in the root meristem (Ioio et al., 2012).
ARR1 -| MIR166	Cytokinin exogenous treatment reduces the expression of MIR165 in the root meristem (Ioio et al., 2012; Hao et al., 2012). In our previous model we incorporated this regulation as CK –| MIR166 (García-Gómez et al., 2017), but we reasoned that this regulation is mediated through the CK signaling pathway.
ARF5 -| MGP	An auxin maximum defines the position of the QC in the endodermal layer of the root meristem (Sabatini et al., 2003). We included this hypothetical negative link between the auxin pathway and the endodermis marker MGP to account for the role of auxin on the decision of becoming endodermis or QC cells. The regulatory network model with this hypothesis recovers the same fixed-point attractors as did the previous version, including a QC and a CEI/Endodermis attractor.
SCR -> WOX5<br>PLT -> WOX5	SCR and PLT form a complex with the Teocinte (CP21) and TCP22 transcriptional regulators. This complex binds to WOX5 promoter and regulates its expression (Shimotohno et al., 2018).

Supplementary Table S1. New interactions included in the updated regulatory network model with respect to the version in García-Gómez and collaborators (2017).

The updated genetic-hormonal regulatory network model recovers 11 fixed-point attractors as did the previous published version of the model (Table S2; García-Gómez et al., 2017). These attractors correlate with the activity configurations observed in different cell types and behaviors along the root meristem, including the cells of the root stem cell niche. It also recovered two cyclic attractors when solved with the synchronic updating scheme. These cyclic attractors emerge due to a mutual inhibition between MIR166 and PHB, and of MGP and WOX5. The activity of these genes do not to oscillate in the root meristem, indicating that these attractors do not represent known cell states of the meristem. These attractors are not recovered by the asynchronic updating scheme nor the continuous model (both acknowledge differences in the updating of the nodes). Thus, the cyclic attractors might be an artifact of the synchronic updating scheme. We aimed to recover only fixed-point attractors so we tested the effect of a positive feedback loop in the regulation of the nodes involved in the cycles: MIR166, PHB, MGP and/or WOX5. We found that a positive feedback on MGP eliminated one of the cyclic attractors. This was accompanied by the appearance of a new fixed-point attractor similar to the endodermis of the transition domain but has no MGP activity.

Node	Logical rule
CK	(PHB **and** **not** ARF) **or** **not** SHR
ARR1	**not** SCR **and** CK
SHY2	ARR1 **and** **not** AUX
AUXIAA	**not** AUX
ARF	**not** AUXIAA
ARF10	**not** (JKD **and** SHR) **and** **not** AUXIAA
ARF5	((PHB **or** PLT) **and** **not** (SHR **and** MGP)) **and** **not** SHY2 **and** **not** AUXIAA
XAL1	ARF
PLT	ARF5 **or** ARF **or** WOX5 **or** XAL1
AUX	1
SCR	SHR **and** JKD **and** SCR
SHR	SHR **or** (SCR **and** JKD)
MIR166	(SCR **and** SHR **and** **not** ARR1) **or** **not** PHB
PHB	((**not** ARR1 **and** PLT) **or** PHB) **and** **not** MIR166
JKD	**not** PHB **and** SHR **and** SCR
MGP	**not** ARF5 **and** SHR **and** SCR **and** MGP
WOX5	**not** ARF10 **and** ARF5 **and** **not** CLE40 **and** SCR **and** PLT
CLE40	**not** SHR

Supplementary Table S2. Logical rules of the root apical meristem regulatory network model.

	CK	ARR1	SHY2	AUX IAA	ARF	ARF10	ARF5	XAL1	PLT	AUX	SCR	SHR	MIR 166	PHB	JKD	MGP	WOX5	CLE40
*QC	0	0	0	0	1	0	1	1	1	1	1	1	1	0	1	0	1	0
*CEI / Endodermis PD	0	0	0	0	1	0	0	1	1	1	1	1	1	0	1	1	0	0
Endodermis TD	0	0	0	1	0	0	0	0	0	0	1	1	1	0	1	1	0	0
*P. Pro-vascular PD	0	0	0	0	1	1	1	1	1	1	0	1	1	0	0	0	0	0
P. Pro-vascular TD	0	0	0	1	0	0	0	0	0	0	0	1	1	0	0	0	0	0
*C. Pro-vascular PD	0	0	0	0	1	1	1	1	1	1	0	1	0	1	0	0	0	0
C. Pro-vascular TD	1	1	1	1	0	0	0	0	0	0	0	1	0	1	0	0	0	0
*C. Pro-vascular TD2	1	1	0	0	1	1	1	1	1	1	0	0	0	1	0	0	0	1
C. Pro-vascular TD3	1	1	1	1	0	0	0	0	0	0	0	0	0	1	0	0	0	1
*Columella 1	1	1	0	0	1	1	1	1	1	1	0	0	1	0	0	0	0	1
Columella 2	1	1	1	1	0	0	0	0	0	0	0	0	1	0	0	0	0	1
Cyclic CP PD	0	0	0	0	1	1	1	1	1	1	0	1	0<->1	0<->1	0	0	0	0
Endodermis TD2	0	0	0	1	0	0	0	0	0	0	1	1	1	0	1	0	0	0

Supplementary Table S3. Attractors recovered by the updated regulatory network model using a synchronic updating scheme. The continuous model recovers only the fixed-point attractors. For the analysis of this paper, we used the attractors recovered with the constitutive activity of auxin (shown with * and in bold). The sum of the activity configurations of these attractors are: QC = 10, CEI =9, P. pro-vascular PD = 8, C. Pro-vascular CP = 8, C. pro-vascular TD2 = 10, Columella = 10.

In the present work we study the cell-fate transition dynamics within the root SCN, where the auxin levels are highest in the root meristem. Thus, we fixed the activity of the node auxin (AUX) to 1 in the regulatory network model. The network model with the AUX node constitutively active recovered 6 fixed-point attractors under both discrete and continuous modelling approaches (shown in * in Table S2). These attractors correspond to the following cells of the root SCN: QC, cortex/endodermis initials (CEI), peripheral pro-vascular initials, central pro-vascular initials, and columella initials; the last attractor describes differentiating cells of the transition domain.

C. Decay rate variation analysis

The following tables, one per initial state (attractor), show the transitions caused by increasing the decay rate of a particular node (indicated above each plot). We plotted the decay rate in the x axis and in the y axis the sum of the activity of all nodes ($\sum_{i=1}^{X} x_i$) in the new attractor reached; this was used to detect sudden changes that may represent attractor transitions. Even when the two attractors yield the same sum, the transition can be seen as noticeable jumps. The final state at which the system converges is indicated at the right. To link an activity configuration to an attractor we expected a 100% similarity between the expected and recovered attractors (expected attractors in Supplementary Table S1). Otherwise, an attractor was associated to an expected cell type as long as the activity of the transcription factors known to underlie cell fate had the correct activity patterns. In the following tables we indicated with an asterisk (*) the cases where the activity of the nodes does not match the activity configuration of an attractor; we also indicate the nodes with a different activity than expected with bold letters. Nodes' activity is reported in the following order: CK, ARR1, SHY2, AUXIAAR, ARFR, ARF10, ARF5, XAL1, PLT, AUX, SCR, SHR, MIR165, PHB, JKD, MGP, WOX5, and CLE40.

Supplementary Table S4. Initial state: QC

ARF5

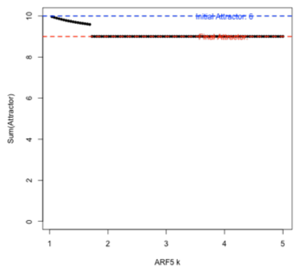

Initial state: 000010111111101010	QC
Final state: 000010011111101100	CEI/Endodermis

ARFr

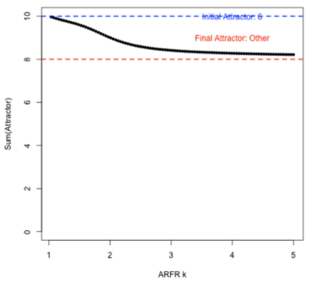

Initial state: 000010111111101010	QC
Final state: 0000**0**01**0**1111101010	QC*

AUX

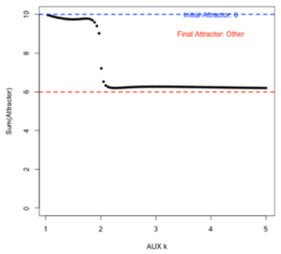

Initial state: 000010111111101010	QC
Final state: 000100000011101100	Endodermis Transition domain

JKD

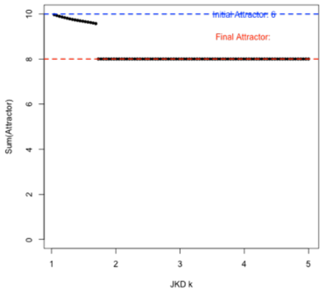

Initial state: 000010111111101010	QC
Final state: 000011111101100000	Peripheral pro-vascular initials

MIR166

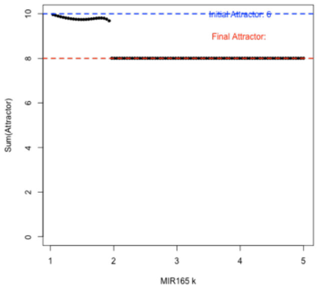

Initial state: 000010111111101010	QC
Final state: 000011111101010000	Central pro-vascular initials

PLT

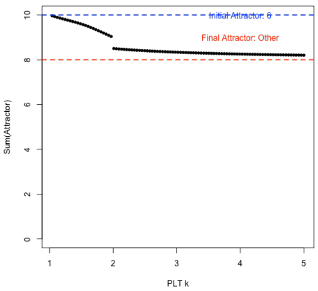

Initial state: 000010111111101010	QC
Final state: 00001001**0**111101100	CEI/Endodermis*

SCR

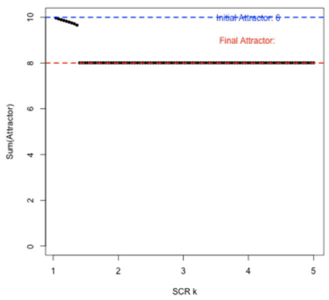

Initial state: 000010111111101010	QC
Final state: 000011111101100000	Peripheral pro-vascular initials

SHR

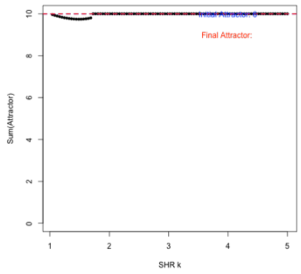

Initial state: 000010111111101010	QC
Final state: 110011111100100001	Columella initials

WOX5

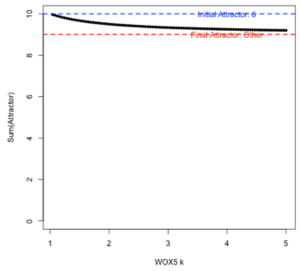

Initial state: 000010111111101010	QC
Final state: 0000101111111010**0**0	QC*

XAL1

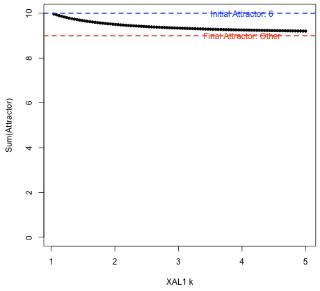

Initial state: 000010111111101010	QC
Final state: 0000101**0**1111101010	QC*

Supplementary Table S5. Initial state: CEI/Endodermis

ARFr

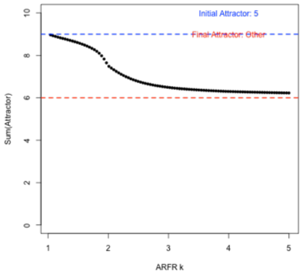

Initial state: 000010011111101100	CEI/Endodermis
Final state: 0000**0**00**00**111101100	CEI/Endodermis*

AUX

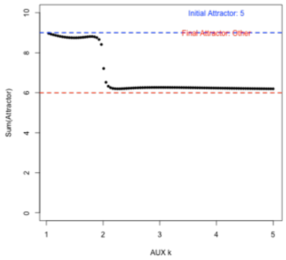

Initial state: 000010011111101100	CEI/Endodermis
Final state: 000100000011101100	Endodermis Transition domain

JKD

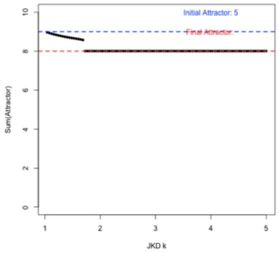

Initial state: 000010011111101100	CEI/Endodermis
Final state: 000011111101100000	Peripheral pro-vascular initials

MGP

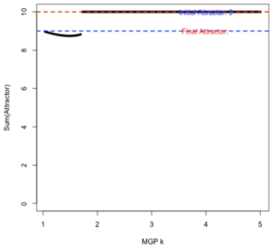

Initial state: 000010011111101100	CEI/Endodermis
Final state: 000010111111101010	QC

MIR166

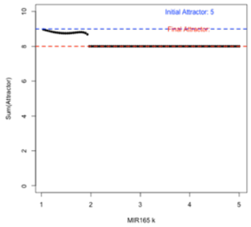

Initial state: 000010011111101100	CEI/Endodermis
Final state: 000011111101010000	Central pro-vascular initials

PLT

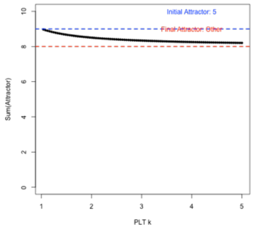

Initial state: 000010011111101100	CEI/Endodermis
Final state: 00001001**0**111101100	CEI/Endodermis*

SCR

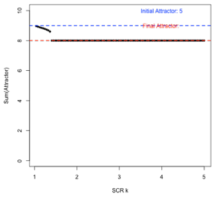

Initial state: 000010011111101100	CEI/Endodermis
Final state: 000011111101100000	Peripheral pro-vascular initials

SHR

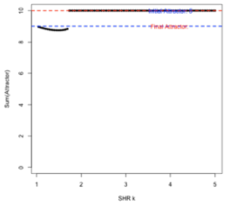

Initial state: 000010011111101100	CEI/Endodermis
Final state: 110011111100100001	Columella initials

XAL1

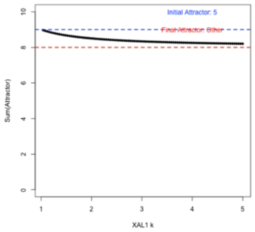

Initial state: 000010011111101100	CEI/Endodermis
Final state: 0000100**0**1111101100	CEI/Endodermis*

Supplementary Table S6. Initial state: Peripheral pro-vascular initials

ARF5

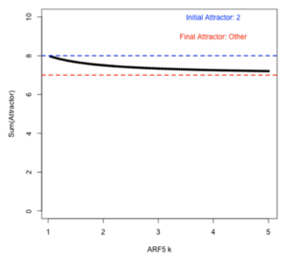

Initial state: 0000111111101100000	Peripheral pro-vascular initials
Final state: 000011**0**111101100000	Peripheral pro-vascular initials*

ARF10

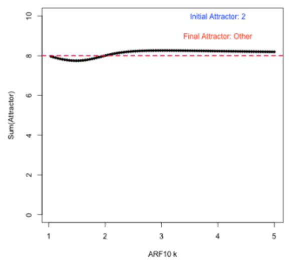

Initial state: 0000111111101100000	Peripheral pro-vascular initials
Final state: 00001**0**11111011000**1**0	Peripheral pro-vascular initials*

ARFr

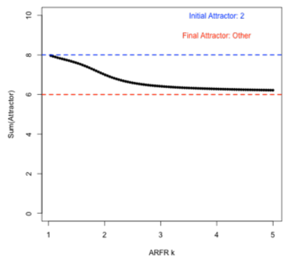

Initial state: 0000111111101100000	Peripheral pro-vascular initials
Final state: 0000**0**11**0**11101100000	Peripheral pro-vascular initials*

AUX

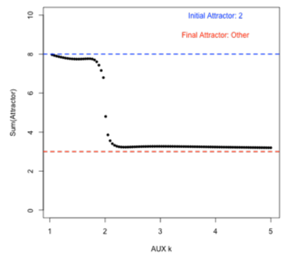

Initial state: 0000111111101100000	Peripheral pro-vascular initials
Final state: 0001000000001100000	Peripheral pro-vascular transition domain

MIR166

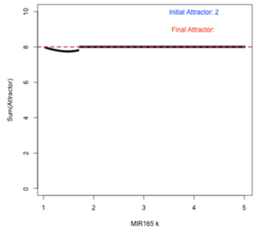

Initial state: 000011111101100000	Peripheral pro-vascular initials
Final state: 000011111101010000	Central pro-vascular initials

PLT

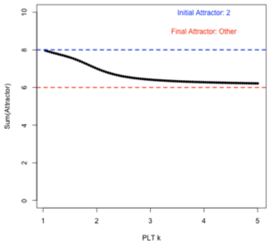

Initial state: 000011111101100000	Peripheral pro-vascular initials
Final state: 000011**0**1**0**101100000	Peripheral pro-vascular initials*

SHR

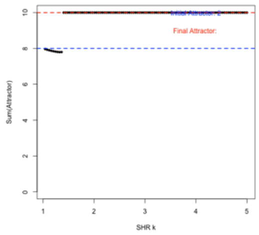

Initial state: 000011111101100000	Peripheral pro-vascular initials
Final state: 110011111100100001	Columella initials

XAL1

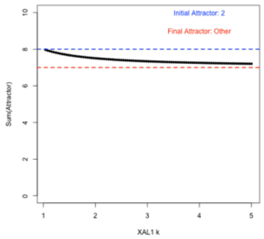

Initial state: 000011111101100000	Peripheral pro-vascular initials
Final state: 0000111**0**1101100000	Peripheral pro-vascular initials*

Supplementary Table S7. Initial state: Central pro-vascular initials

ARF5

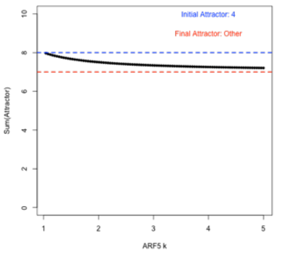

Initial state: 000011111101010000	Central pro-vascular initials
Final state: 000011**0**11101010000	Central pro-vascular initials*

ARF10

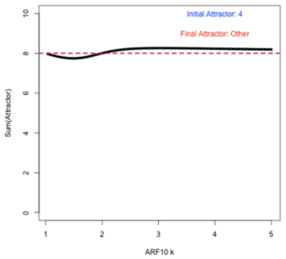

Initial state: 000011111101010000	Central pro-vascular initials
Final state: 00001**0**1111010100**1**0	Central pro-vascular initials*

ARFr

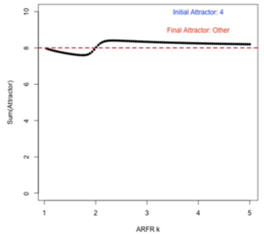

Initial state: 000011111101010000	Central pro-vascular initials
Final state: **11**00**0**11**0**1101010000	Central pro-vascular *

AUX

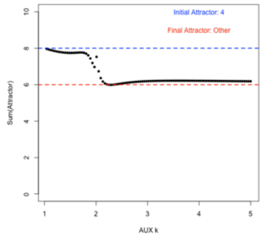

Initial state: 000011111101010000	Central pro-vascular initials
Final state: 111100000001010000	Central pro-vascular transition domain*

PHB

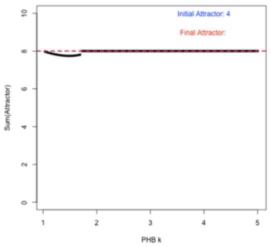

Initial state: 000011111101010000	Central pro-vascular initials
Final state: 000011111101100000	Peripheral pro-vascular initials

PLT

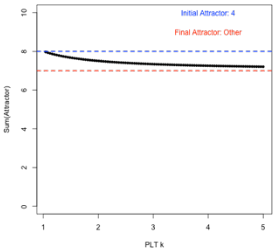

Initial state: 000011111101010000	Central pro-vascular initials
Final state: 00001111**0**101010000	Central pro-vascular initials

SHR

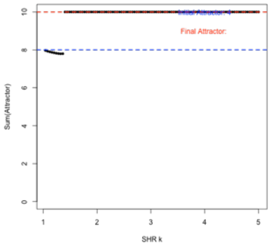

Initial state: 000011111101010000	Central pro-vascular initials
Final state: 110011111100010001	Transition domain

XAL1

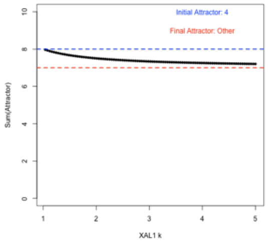

Initial state: 000011111101010000	Central pro-vascular initials
Final state: 0000111**0**1101010000	Central pro-vascular initials*

Supplementary Table S8. Initial state: Columella initials

ARF5

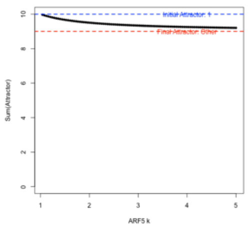

Initial state: 110011111100100001	Columella initials
Final state: 110011**0**11100100001	Columella initials*

ARF10

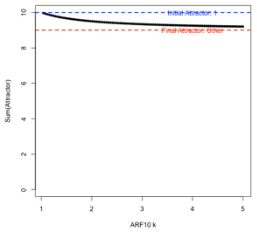

Initial state: 110011111100100001	Columella initials
Final state: 11001**0**111100100001	Columella initials*

ARFr

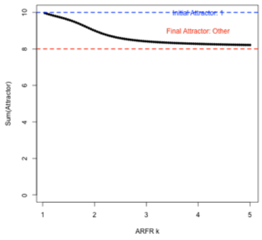

Initial state: 110011111100100001	Columella initials
Final state: 1100**0**11**0**1100100001	Columella initials*

ARR1

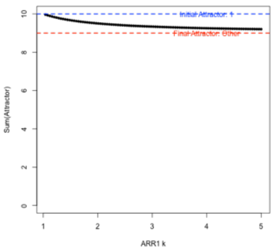

Initial state: 110011111100100001	Columella initials
Final state: 1**0**0011111100100001	Columella initials*

AUX

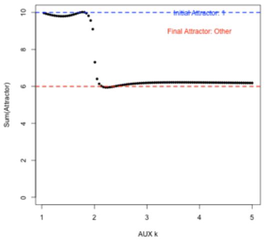

Initial state: 110011111100100001	Columella initials
Final state: 111100000000100001	Columella initials

CK

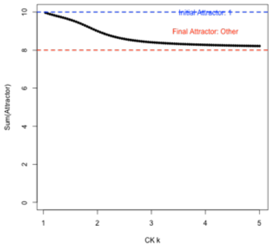

Initial state: 110011111100100001	Columella initials
Final state: **00**0011111100100001	Columella initials*

CLE40

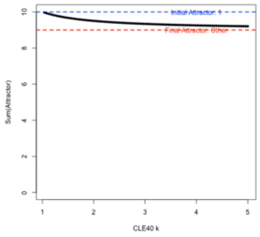

Initial state: 110011111100100001	Columella initials
Final state: 110011111100100000	Columella initials*

MIR166

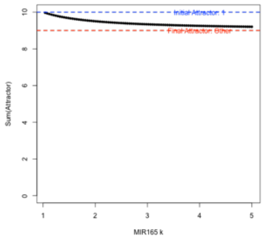

Initial state: 110011111100100001	Columella initials
Final state: 110011111100**0**00001	Columella initials*

PLT

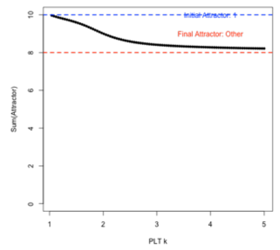

Initial state: 110011111100100001	Columella initials
Final state: 110011**0**1**0**100100001	Columella initials*

XAL1

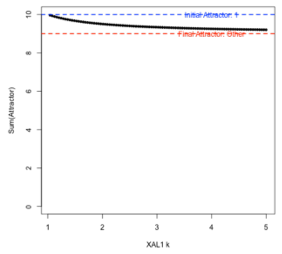

Initial state: 110011111100100001	Columella initials
Final state: 1100111**0**1100100001	Columella initials*

Supplementary Table S9. Initial state: Transition domain

ARF5

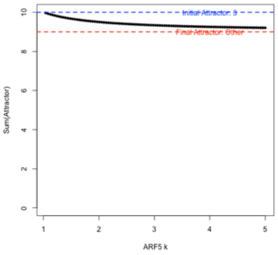

Initial state: 110011111100010001	Transition domain
Final state: 110011**0**11100010001	Transition domain*

ARF10

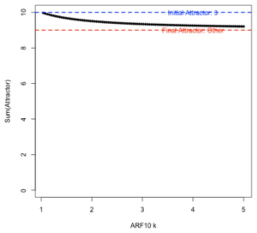

Initial state: 110011111100010001	Transition domain
Final state: 110010111100010001	Transition domain*

ARFr

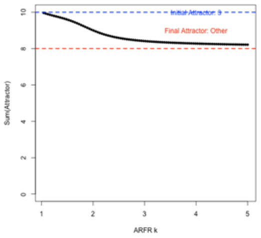

Initial state: 110011111100010001	Transition domain
Final state: 1100**0**11**0**1100010001	Transition domain*

ARR1

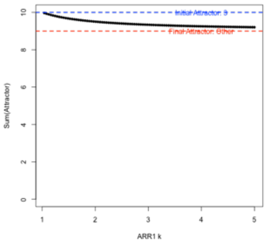

Initial state: 110011111100010001	Transition domain
Final state: 1**0**0011111100010001	Transition domain*

AUX

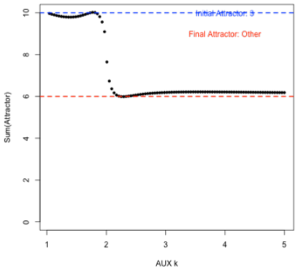

Initial state: 110011111100010001	Transition domain
Final state: 11110000000010001	Transition domain

CK

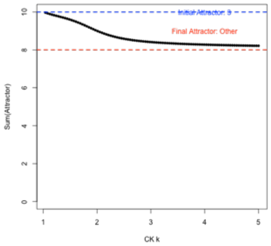

Initial state: 11001111100010001	Transition domain
Final state: **00**0011111100010001	Transition domain*

CLE40

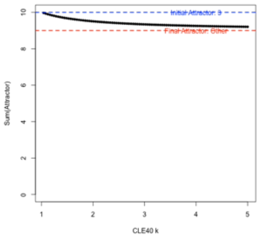

Initial state: 11001111100010001	Transition domain
Final state: 1100111110001000**0**	Transition domain*

PHB

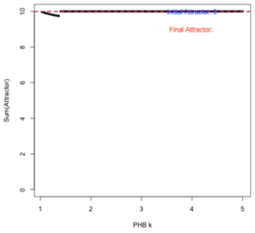

Initial state: 11001111100010001	Transition domain
Final state: 11001111100100001	Columella initials

PLT

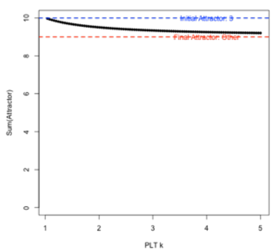

Initial state: 110011111100010001	Transition domain
Final state: 11001111**0**100010001	Transition domain*

XAL1

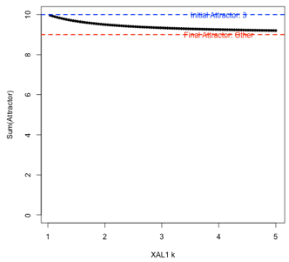

Initial state: 110011111100010001	Transition domain
Final state: 1100111**0**1100010001	Transition domain*

D. Parameter *h* Variation

The parameter *h* determines the strength of the interactions and controls if the activation curve of the nodes resembles a step function, a logistic function or a straight line. We performed the decay rate analysis with three different *h* values (10, 50 and 100), the same for all nodes. The attractor transitions caused by increasing the decay rate of a particular node are the same with the three *h* analysed. We also performed simulations where each node has a different *h* (within the range 10-50), and again the transitions were the same. The results are presented in the following tables, one per initial state (attractor).

Supplementary Table S10. Initial state: QC

ARF5

h = 10	h = 50	h = 100
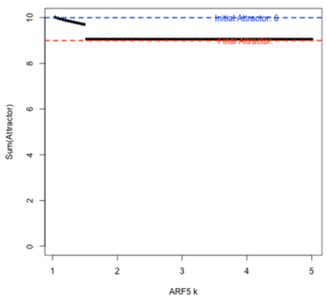	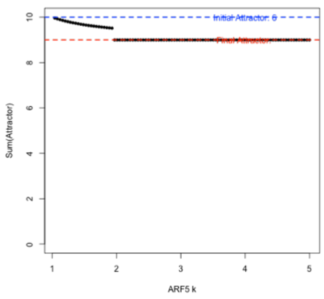	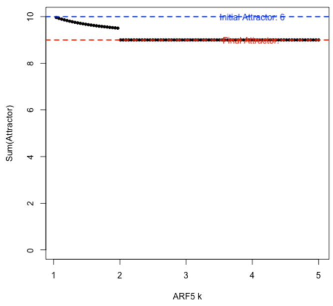

ARF

h = 10	h = 50	h = 100
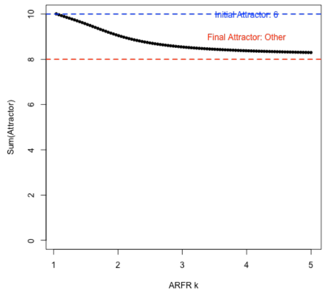	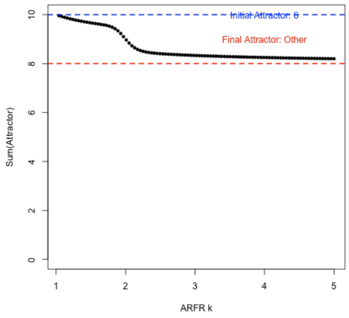	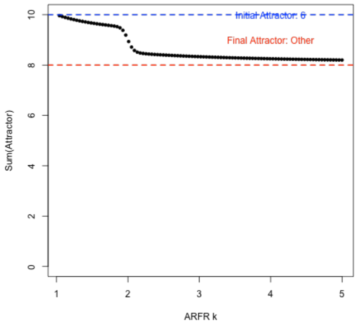

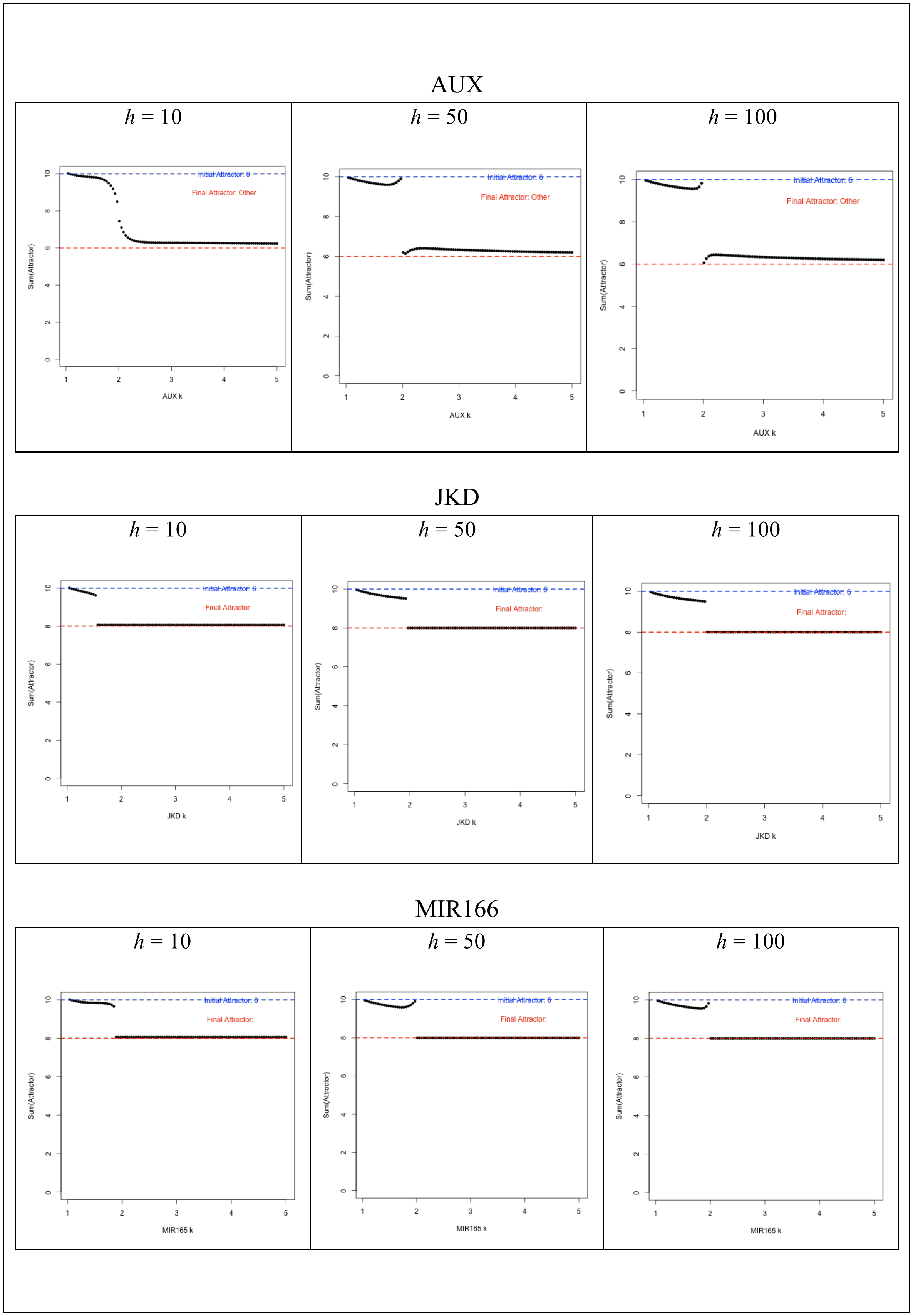

AUX
h = 10
h = 50
h = 100
Initial Attractor: 6
Final Attractor: Other
Sum(Attractor)
AUX k
JKD
h = 10
h = 50
h = 100
Initial Attractor: 6
Final Attractor:
Sum(Attractor)
JKD k
MIR166
h = 10
h = 50
h = 100
Initial Attractor: 6
Final Attractor:
Sum(Attractor)
MIR165 k

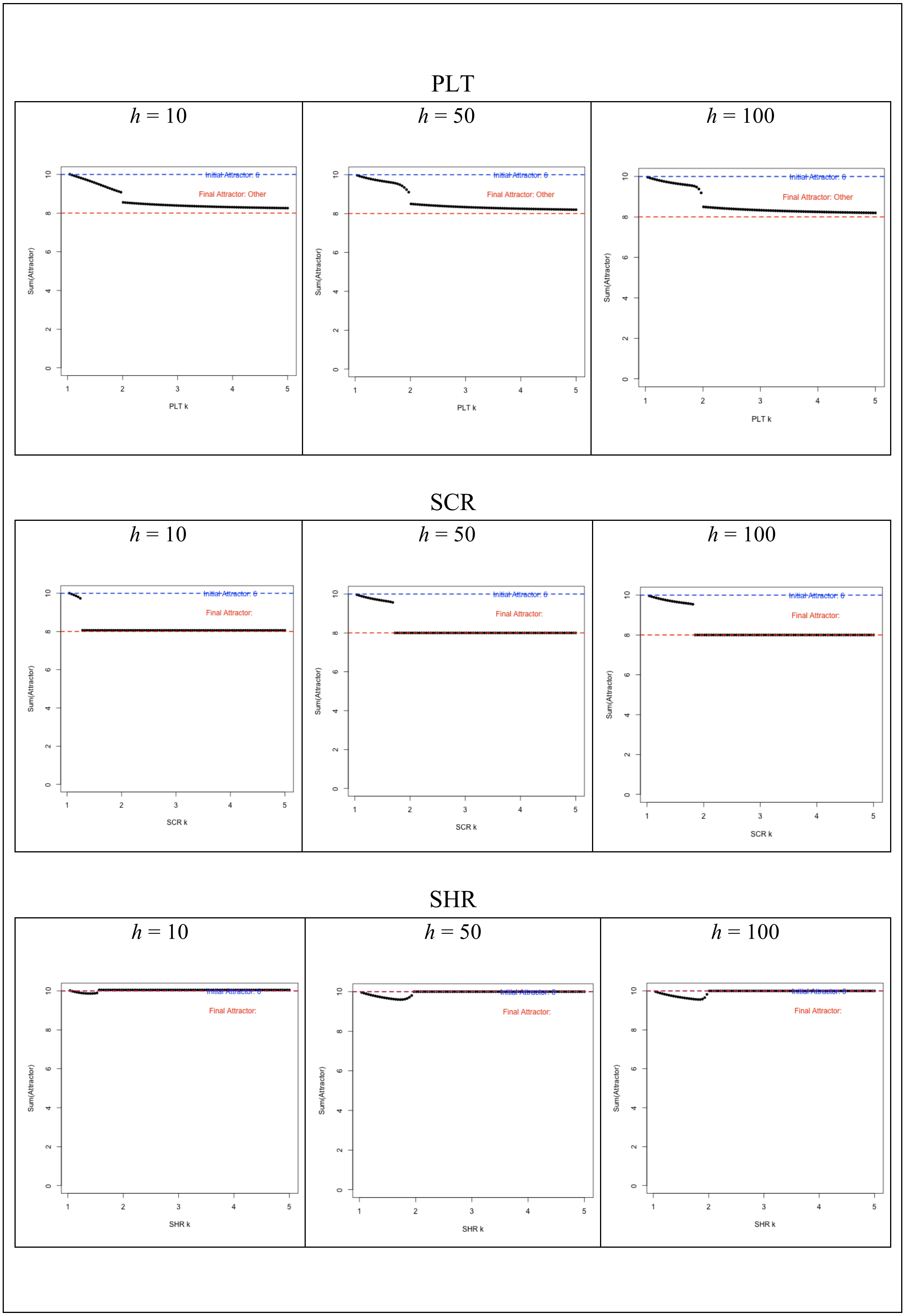
PLT
h = 10
h = 50
h = 100
Sum(Attractor)
Initial Attractor: 6
Final Attractor: Other
PLT k
SCR
h = 10
h = 50
h = 100
Sum(Attractor)
Initial Attractor: 6
Final Attractor:
SCR k
SHR
h = 10
h = 50
h = 100
Sum(Attractor)
Initial Attractor: 6
Final Attractor:
SHR k

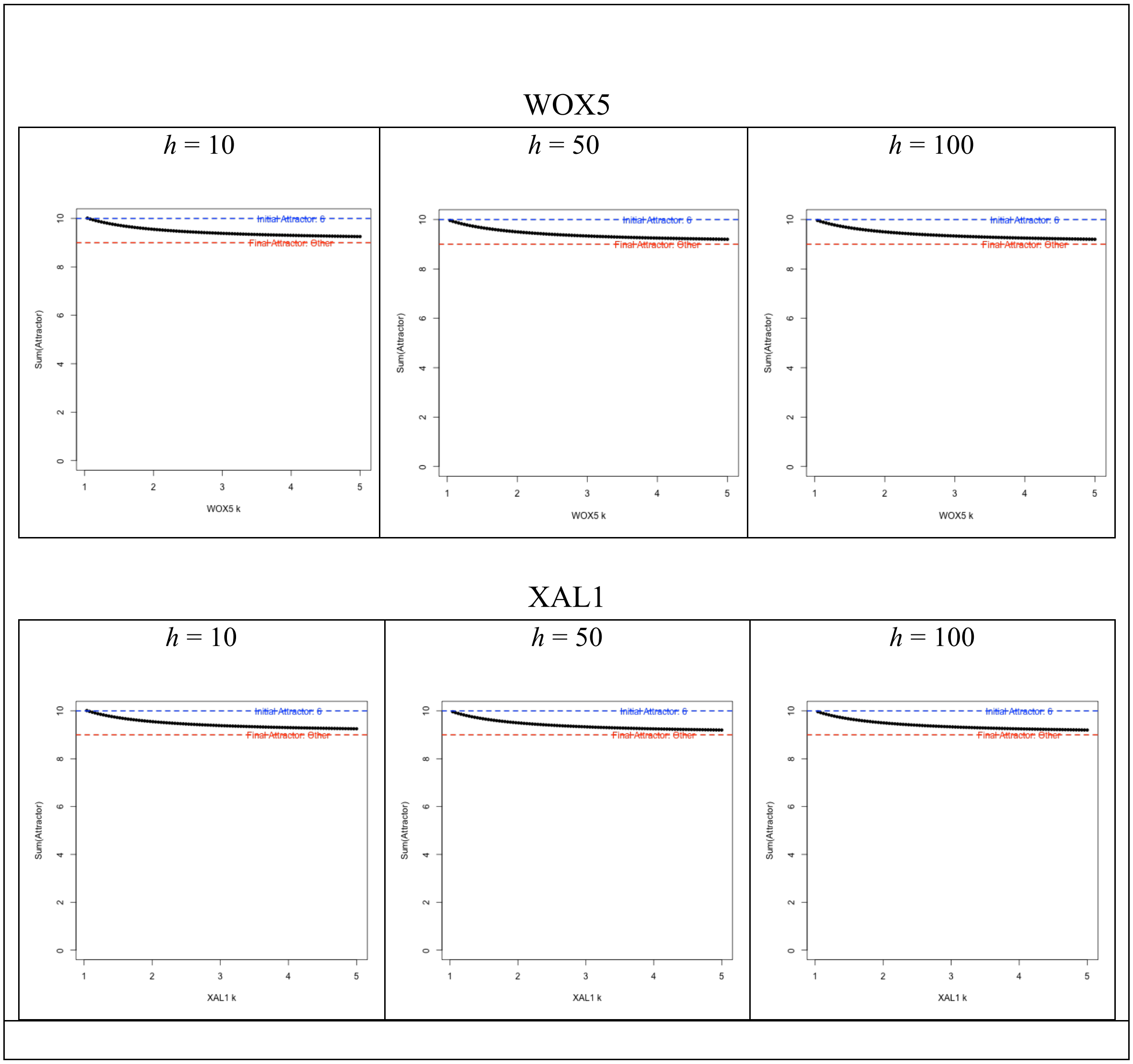

WOX5
h = 10
h = 50
h = 100
Initial Attractor: 6
Final Attractor: Other
Sum(Attractor)
WOX5 k
XAL1
h = 10
h = 50
h = 100
Initial Attractor: 6
Final Attractor: Other
Sum(Attractor)
XAL1 k

Supplementary Table S11. Initial state: CEI/Endodermis

ARFr

h = 10

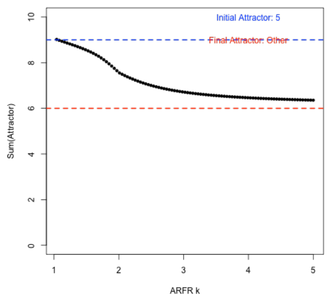

h = 50

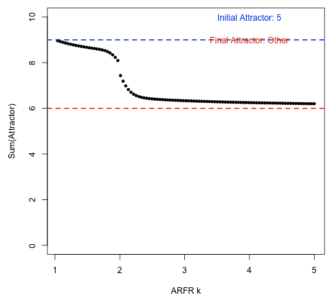

h = 100

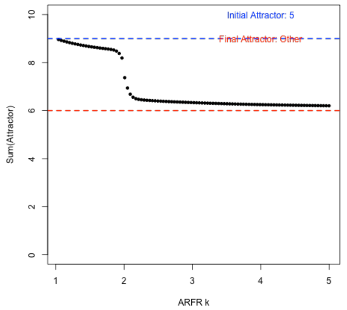

AUX

h = 10

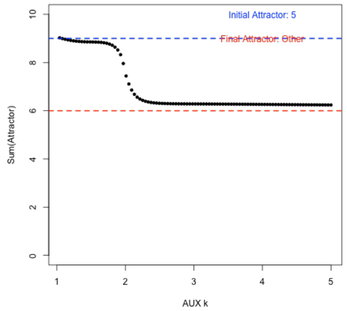

h = 50

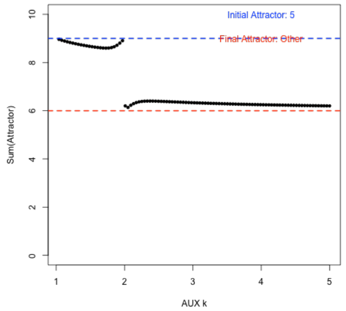

h = 100

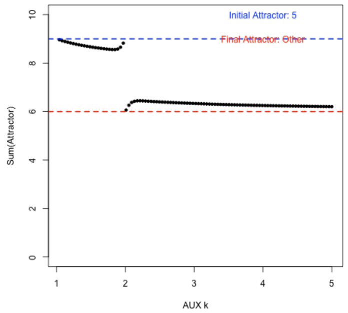

JKD

h = 10

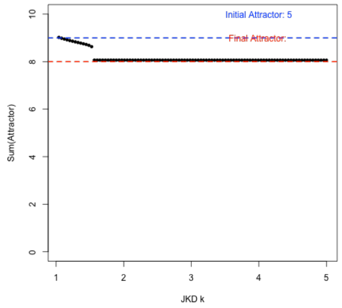

h = 50

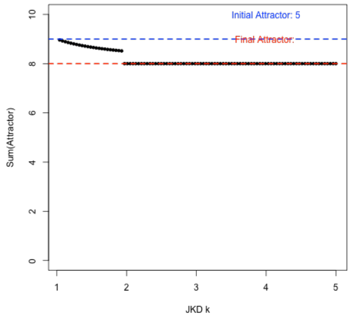

h = 100

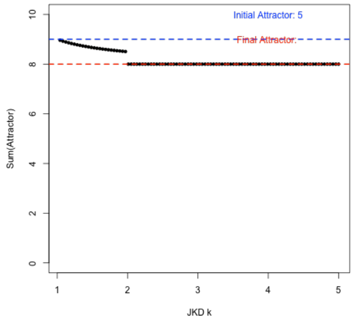

MGP

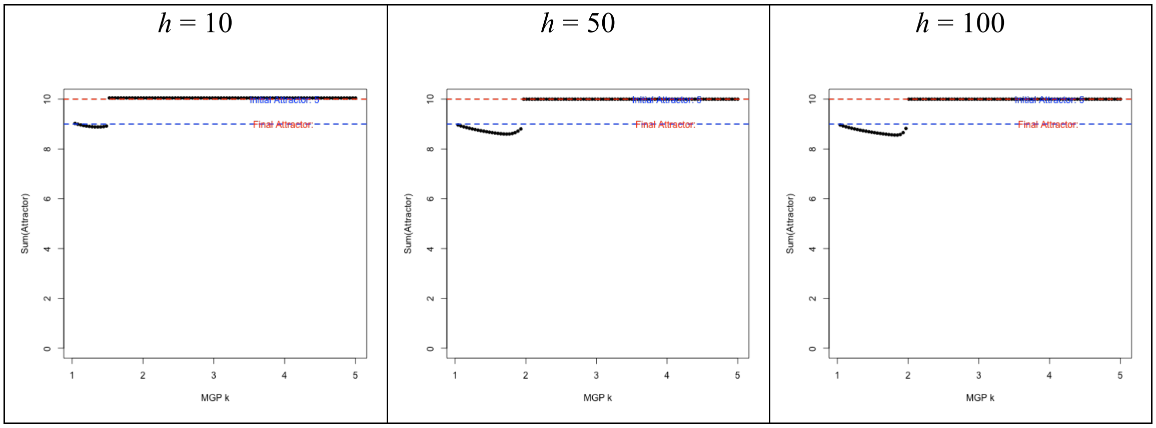

MIR166

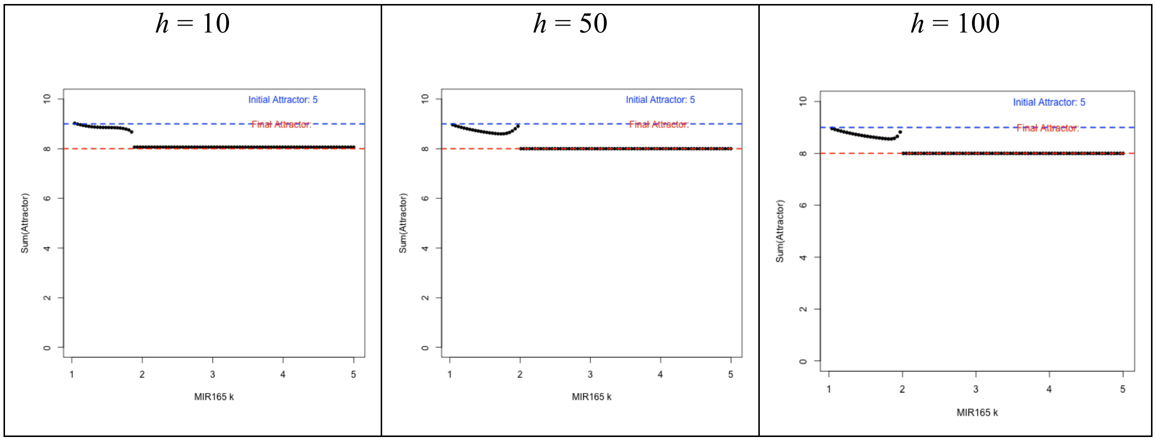

PLT

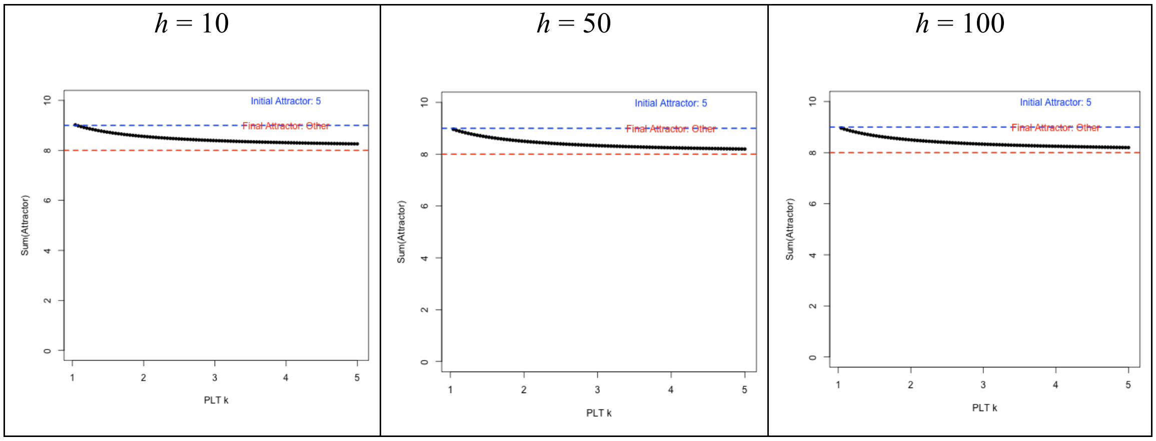

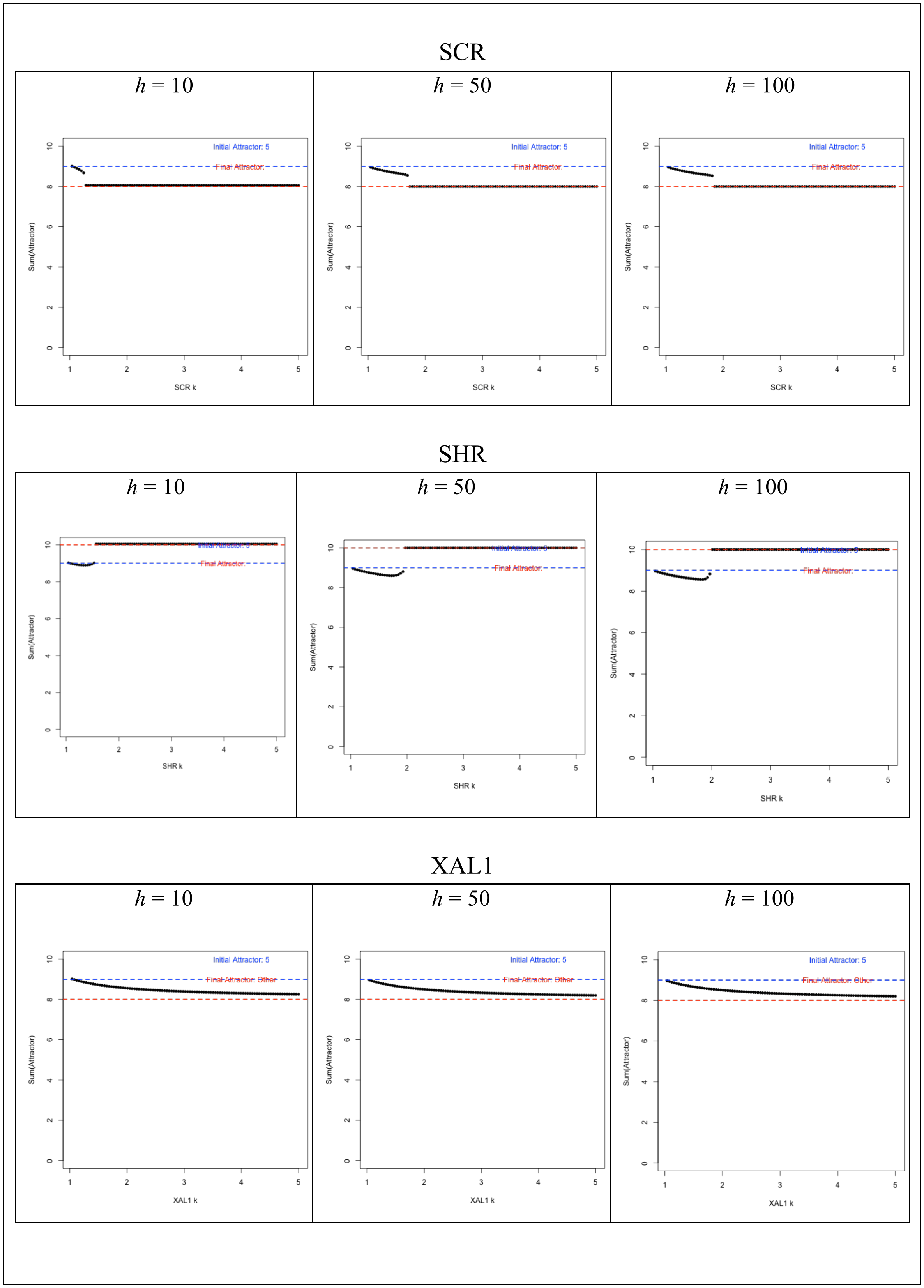
SCR
h = 10
h = 50
h = 100
Sum(Attractor)
Initial Attractor: 5
Final Attractor:
SCR k
SHR
h = 10
h = 50
h = 100
Sum(Attractor)
Initial Attractor: 5
Final Attractor:
SHR k
XAL1
h = 10
h = 50
h = 100
Sum(Attractor)
Initial Attractor: 5
Final Attractor: Other
XAL1 k

Supplementary Table S12. Initial state: Peripheral pro-vascular initials

ARF5

$h = 10$

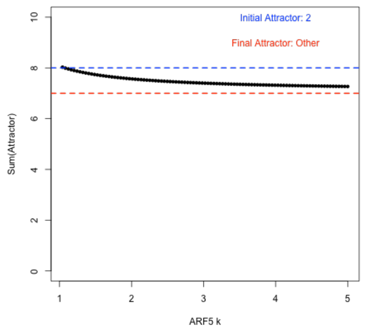

$h = 50$

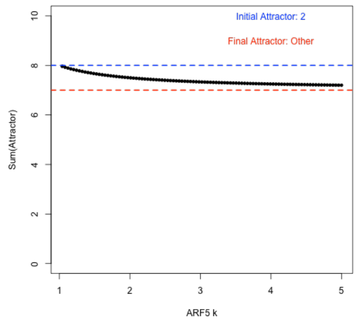

$h = 100$

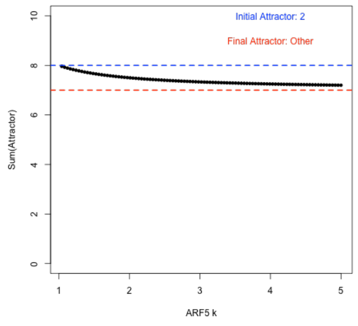

ARF10

$h = 10$

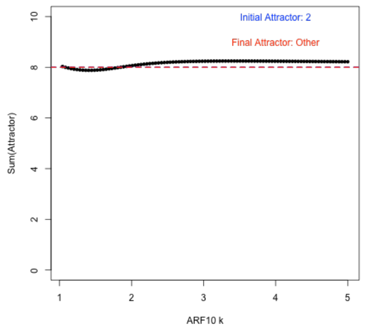

$h = 50$

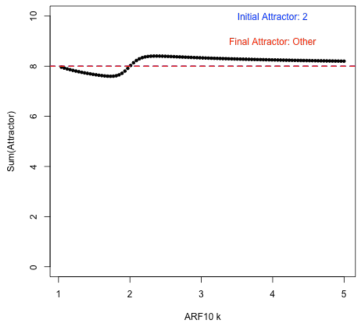

$h = 100$

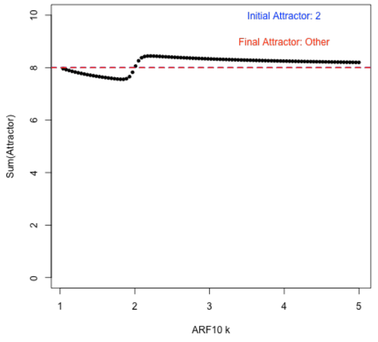

ARFr

$h = 10$

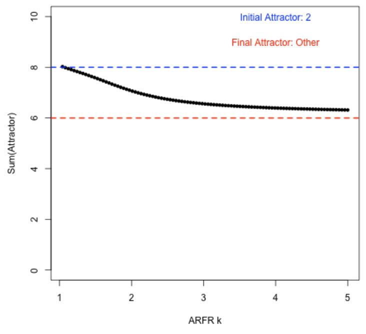

$h = 50$

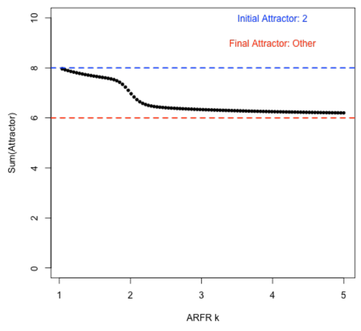

$h = 100$

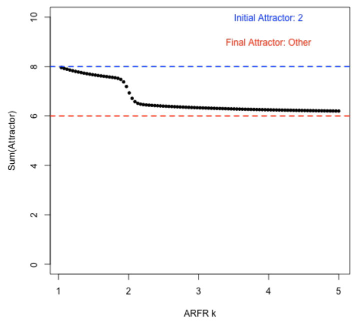

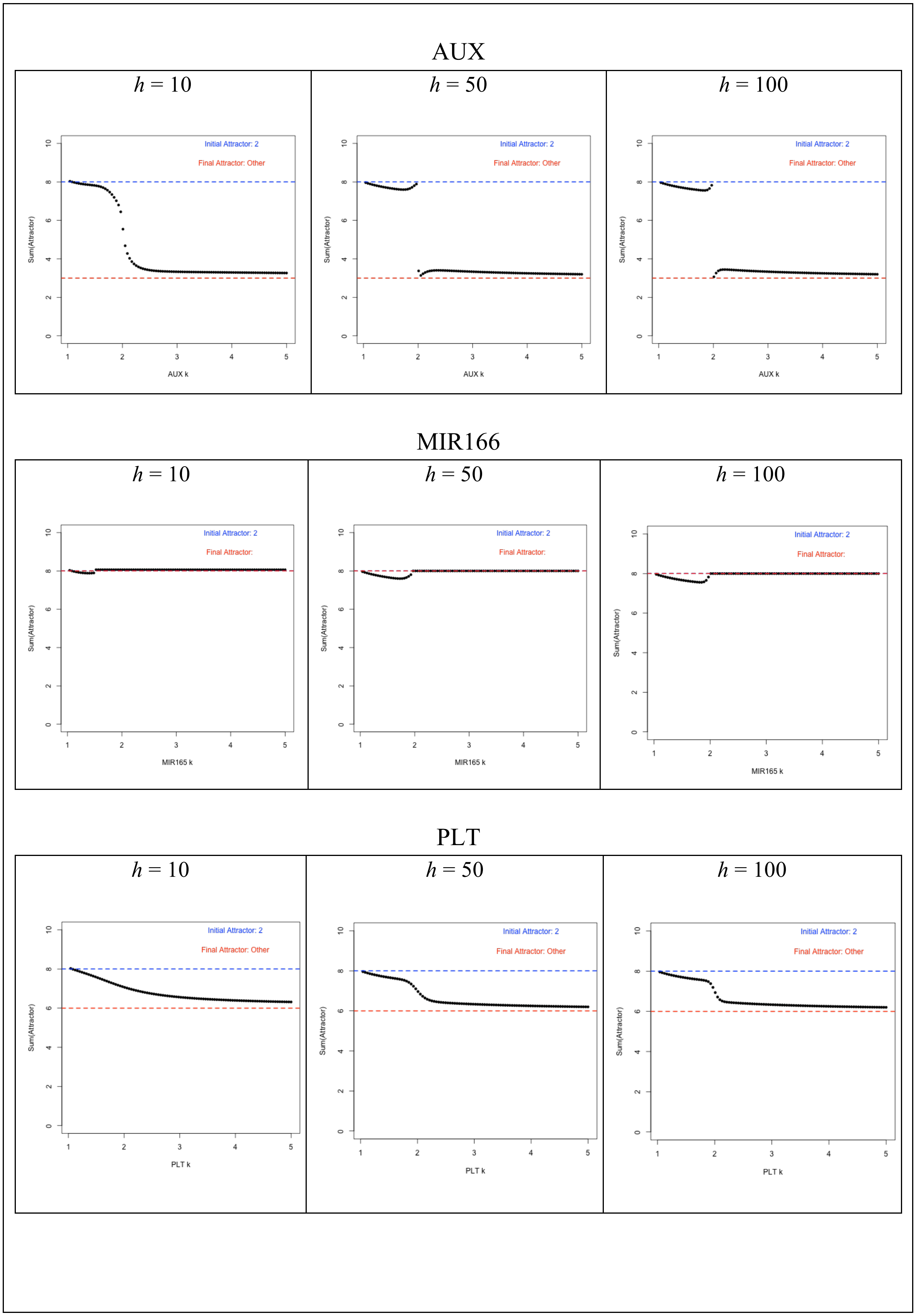
AUX
h = 10
h = 50
h = 100
Initial Attractor: 2
Final Attractor: Other
Sum(Attractor)
AUX k
MIR166
h = 10
h = 50
h = 100
Initial Attractor: 2
Final Attractor:
Sum(Attractor)
MIR165 k
PLT
h = 10
h = 50
h = 100
Initial Attractor: 2
Final Attractor: Other
Sum(Attractor)
PLT k

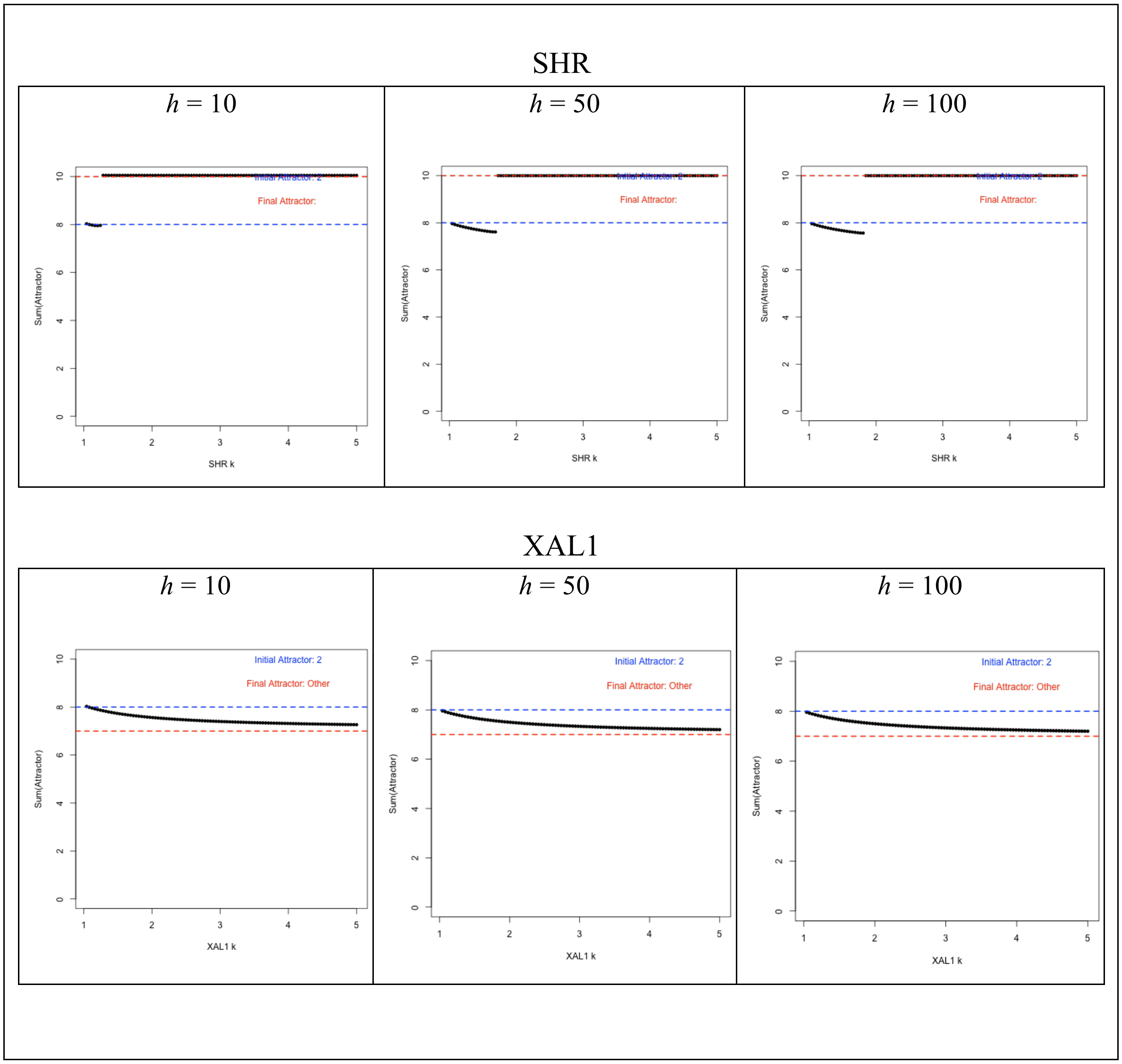
SHR
h = 10
h = 50
h = 100
Initial Attractor: 2
Final Attractor:
Sum(Attractor)
SHR k
XAL1
h = 10
h = 50
h = 100
Initial Attractor: 2
Final Attractor: Other
Sum(Attractor)
XAL1 k

Supplementary Table S13. Initial state: Central pro-vascular initials

ARF5

$h = 10$	$h = 50$	$h = 100$
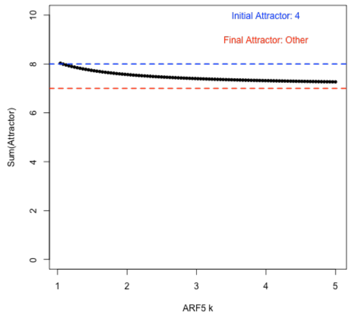	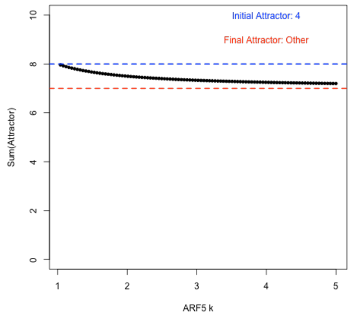	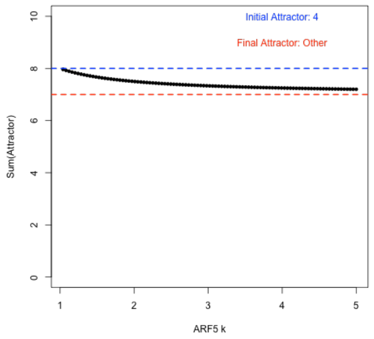

ARF10

$h = 10$	$h = 50$	$h = 100$
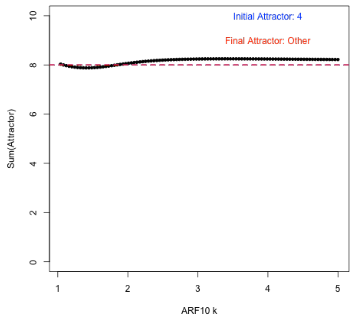	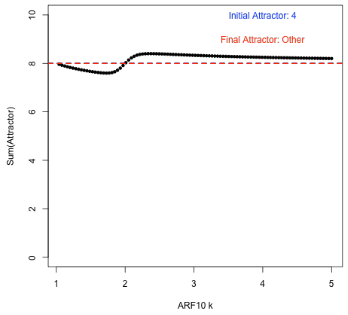	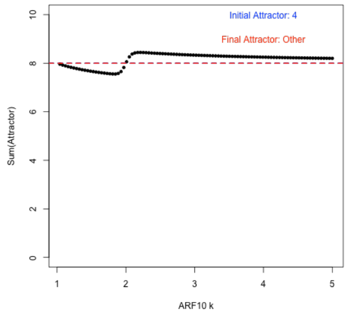

ARFr

$h = 10$	$h = 50$	$h = 100$
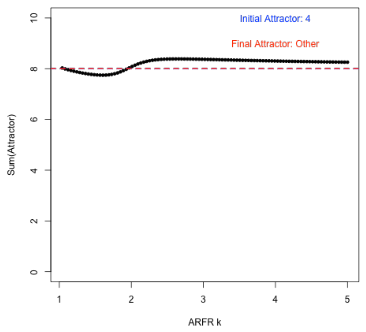	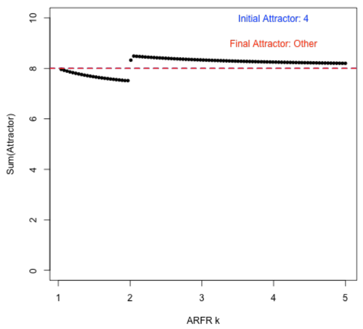	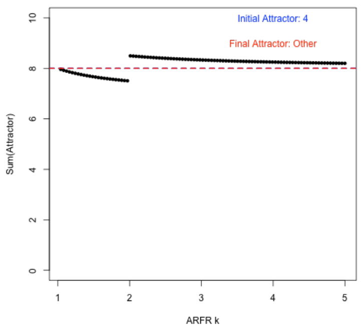

AUX

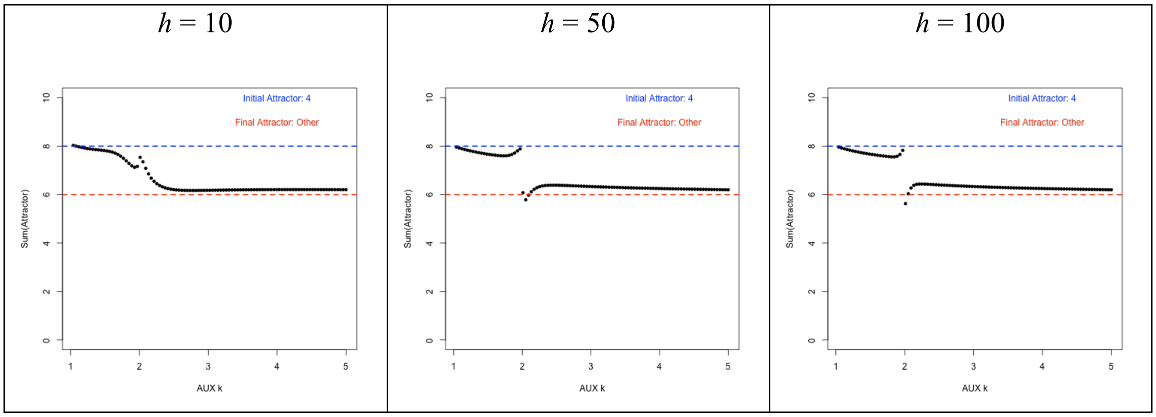
h = 10
h = 50
h = 100
Initial Attractor: 4
Final Attractor: Other
Sum(Attractor)
AUX k

PHB

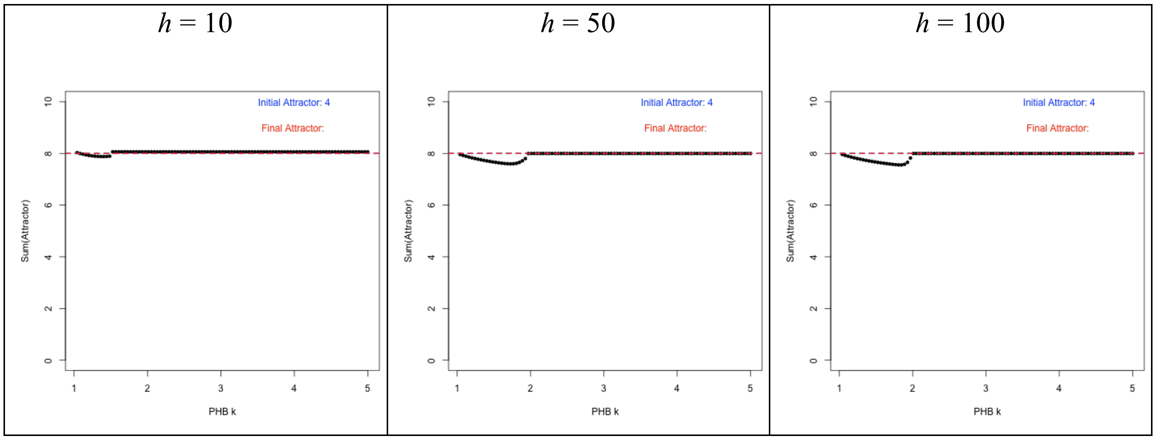
h = 10
h = 50
h = 100
Initial Attractor: 4
Final Attractor:
Sum(Attractor)
PHB k

PLT

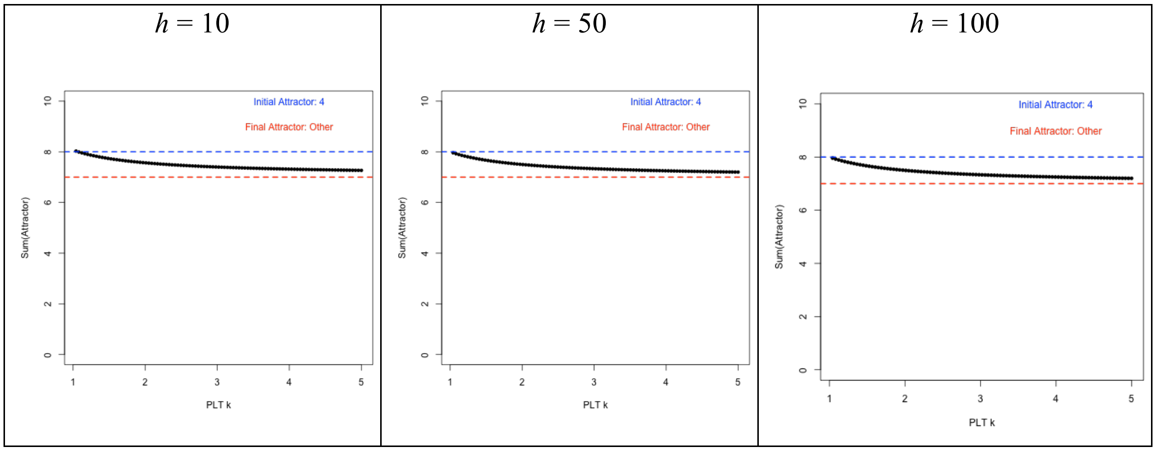
h = 10
h = 50
h = 100
Initial Attractor: 4
Final Attractor: Other
Sum(Attractor)
PLT k

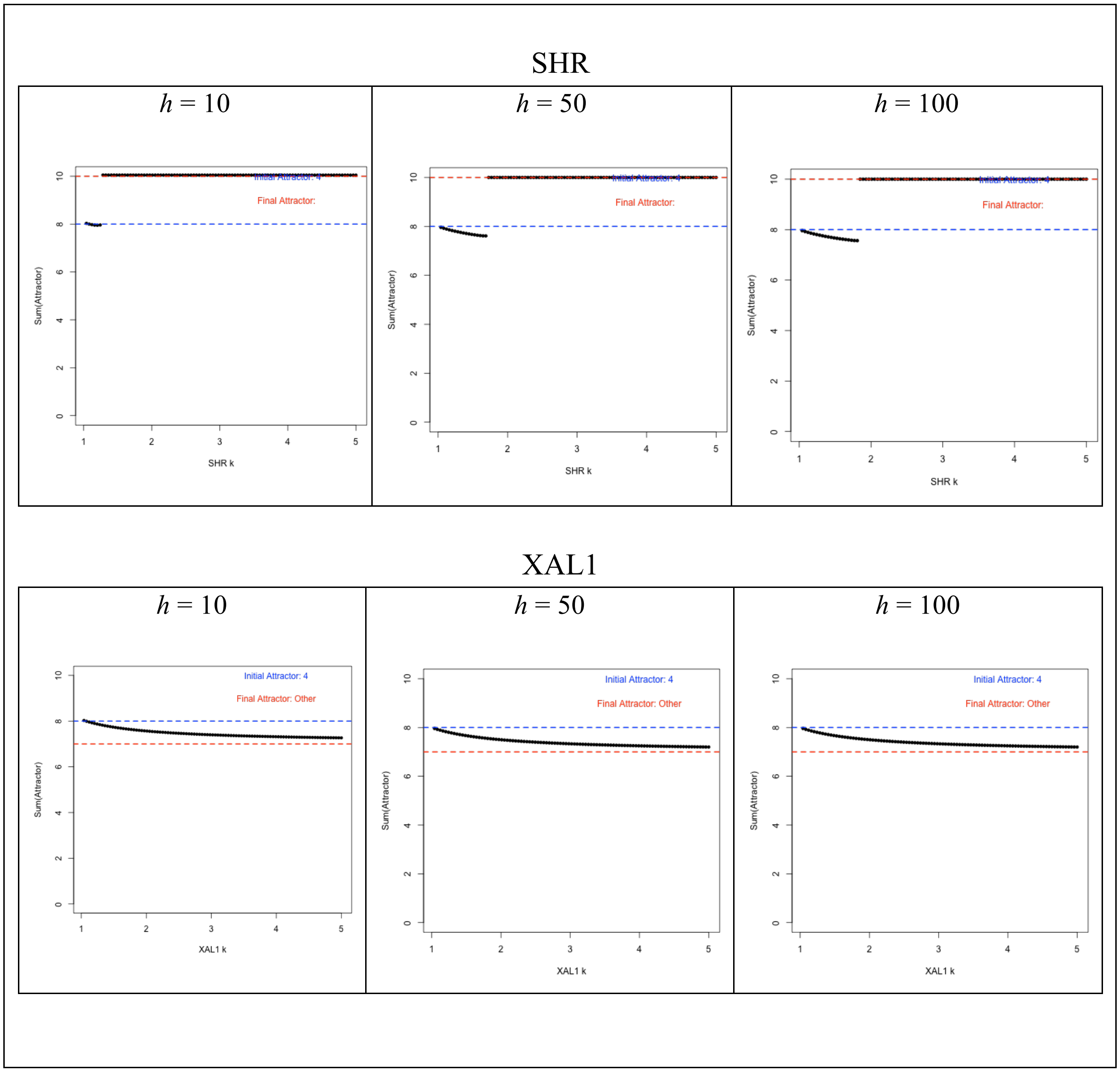
SHR
h = 10
h = 50
h = 100
Final Attractor:
Sum(Attractor)
SHR k
XAL1
h = 10
h = 50
h = 100
Initial Attractor: 4
Final Attractor: Other
Sum(Attractor)
XAL1 k

Supplementary Table S14. Initial state: Columella initials

ARF5

h = 10

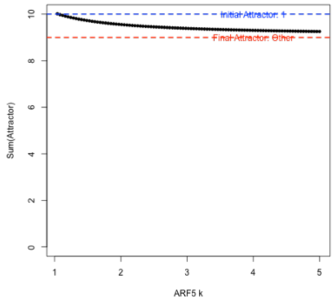

h = 50

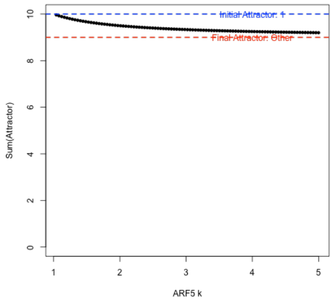

h = 100

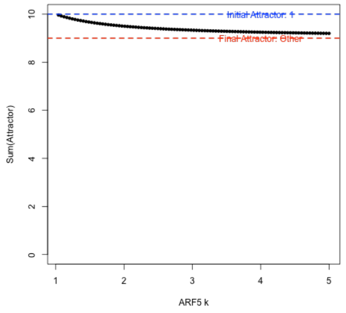

ARF10

h = 10

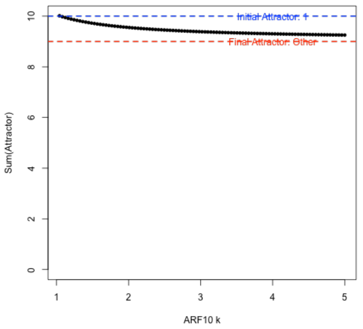

h = 50

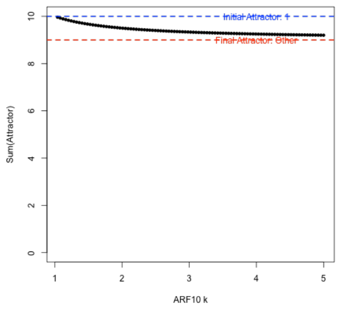

h = 100

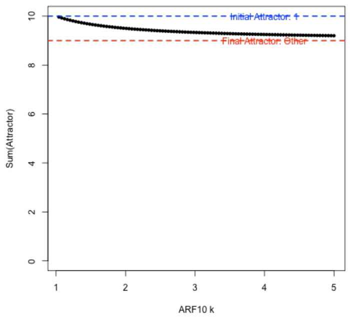

ARFr

h = 10

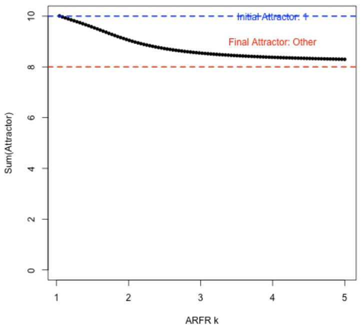

h = 50

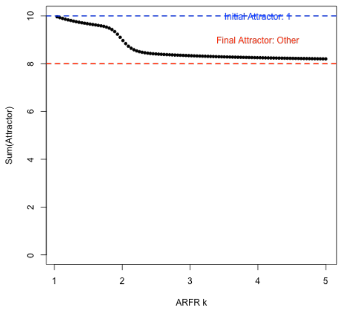

h = 100

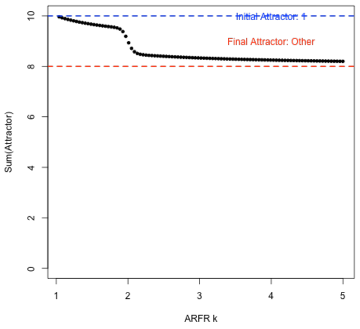

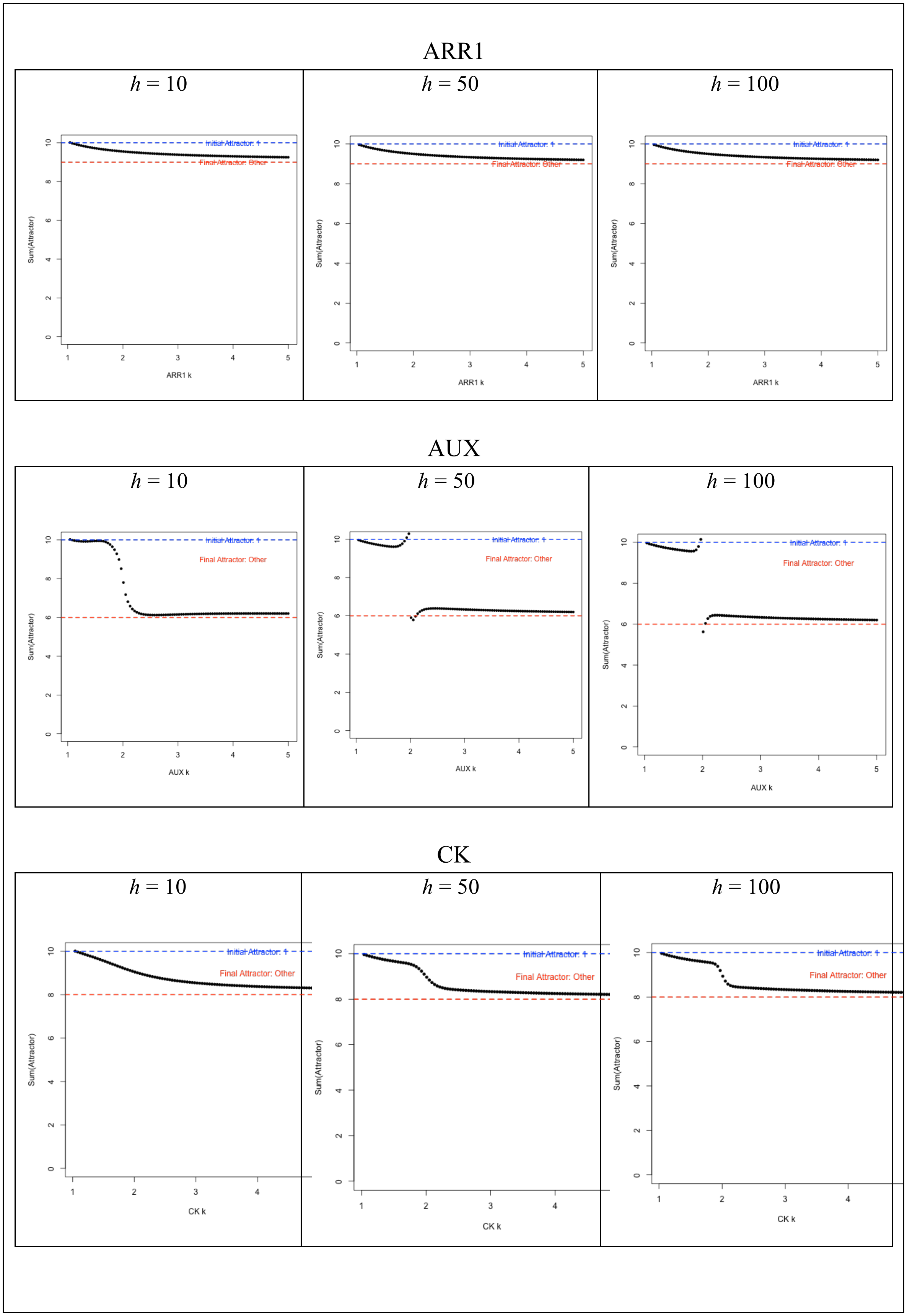
ARR1
h = 10
h = 50
h = 100
Initial Attractor: 1
Final Attractor: Other
Sum(Attractor)
ARR1 k
AUX
h = 10
h = 50
h = 100
Initial Attractor: 1
Final Attractor: Other
Sum(Attractor)
AUX k
CK
h = 10
h = 50
h = 100
Initial Attractor: 1
Final Attractor: Other
Sum(Attractor)
CK k

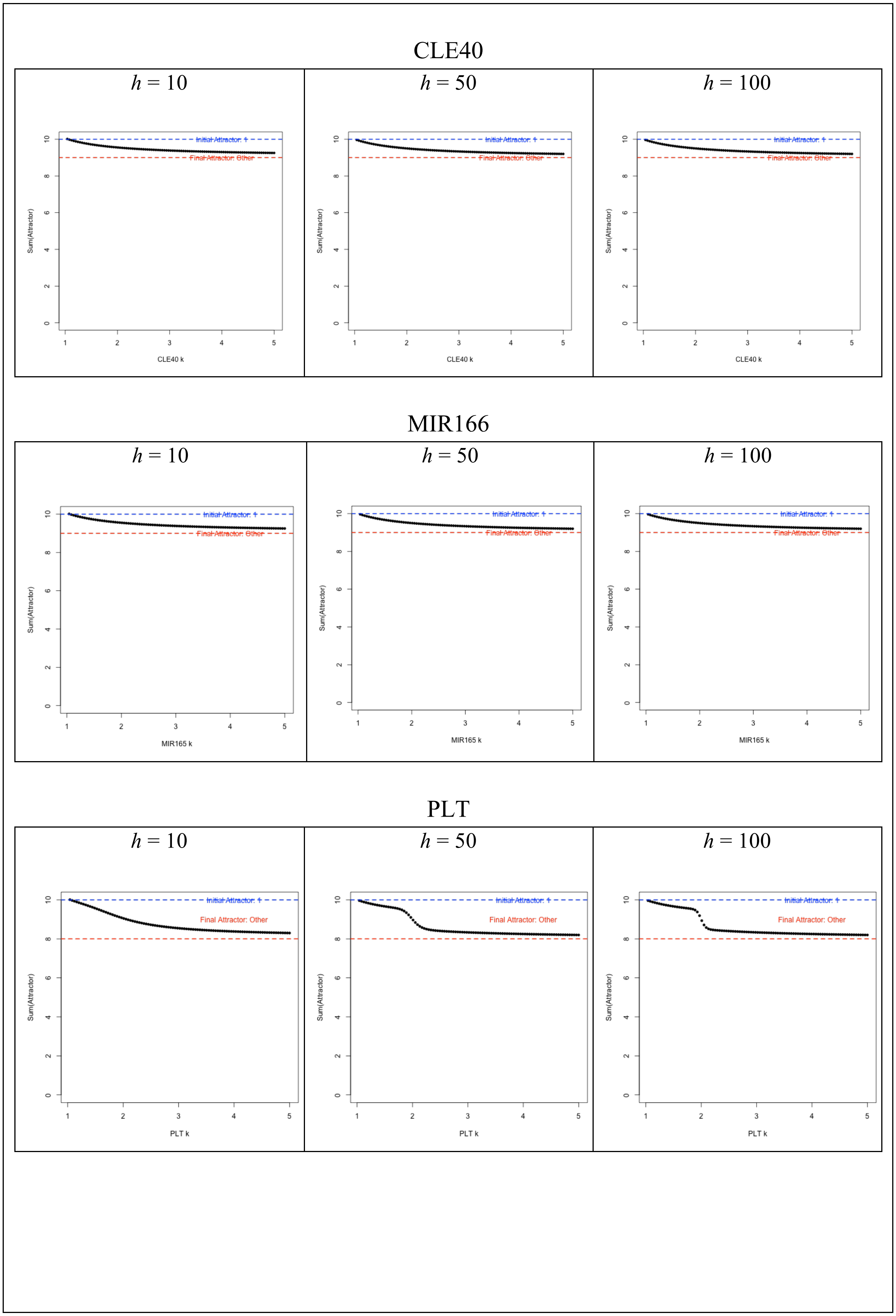

CLE40
h = 10
h = 50
h = 100
Sum(Attractor)
Initial Attractor: 1
Final Attractor: Other
CLE40 k
MIR166
h = 10
h = 50
h = 100
Sum(Attractor)
Initial Attractor: 1
Final Attractor: Other
MIR165 k
PLT
h = 10
h = 50
h = 100
Sum(Attractor)
Initial Attractor: 1
Final Attractor: Other
PLT k

XAL1

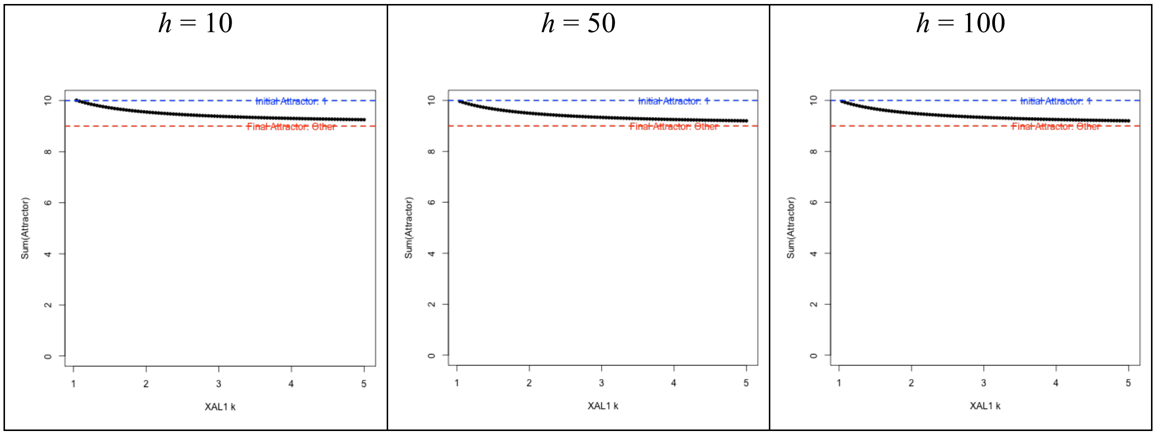
h = 10
h = 50
h = 100
Initial Attractor: 1
Final Attractor: Other
Sum(Attractor)
XAL1 k

Supplementary Table S15. Initial state: Transition domain

ARF5

h = 10	*h* = 50	*h* = 100
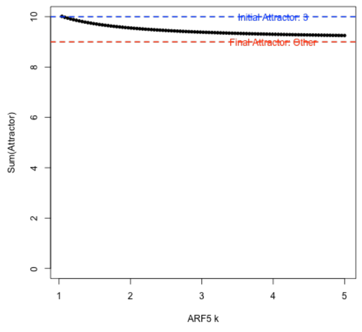	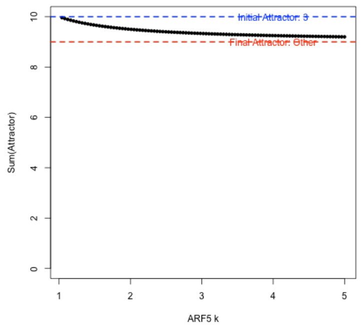	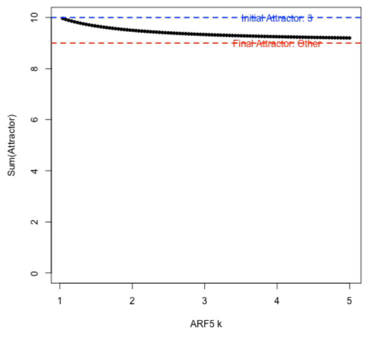

ARF10

h = 10	*h* = 50	*h* = 100
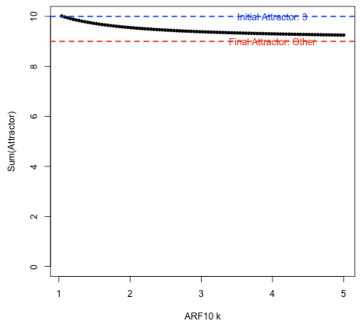	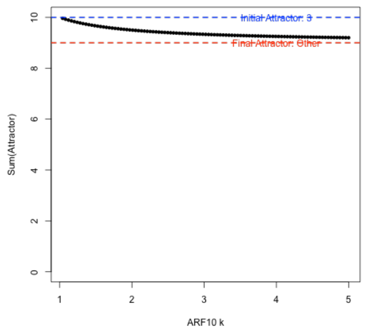	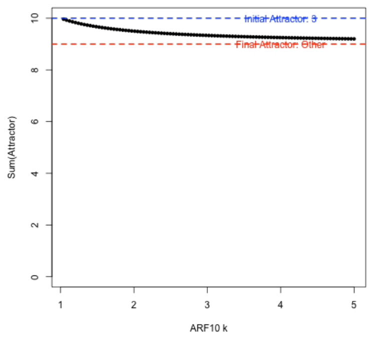

ARFr

h = 10	*h* = 50	*h* = 100
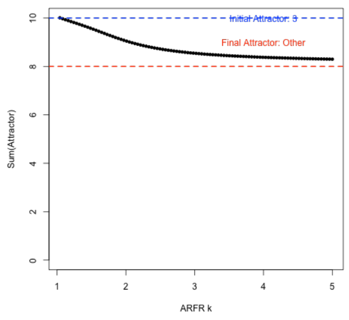	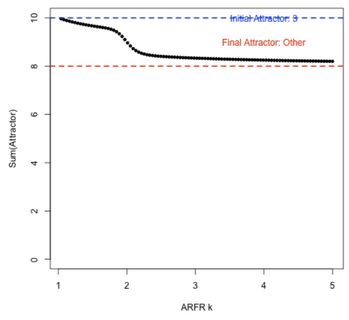	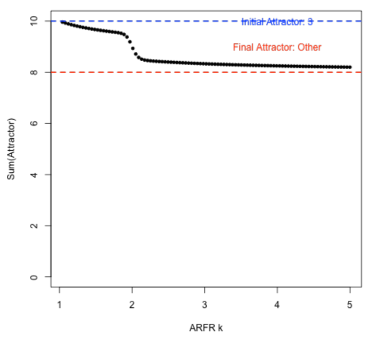

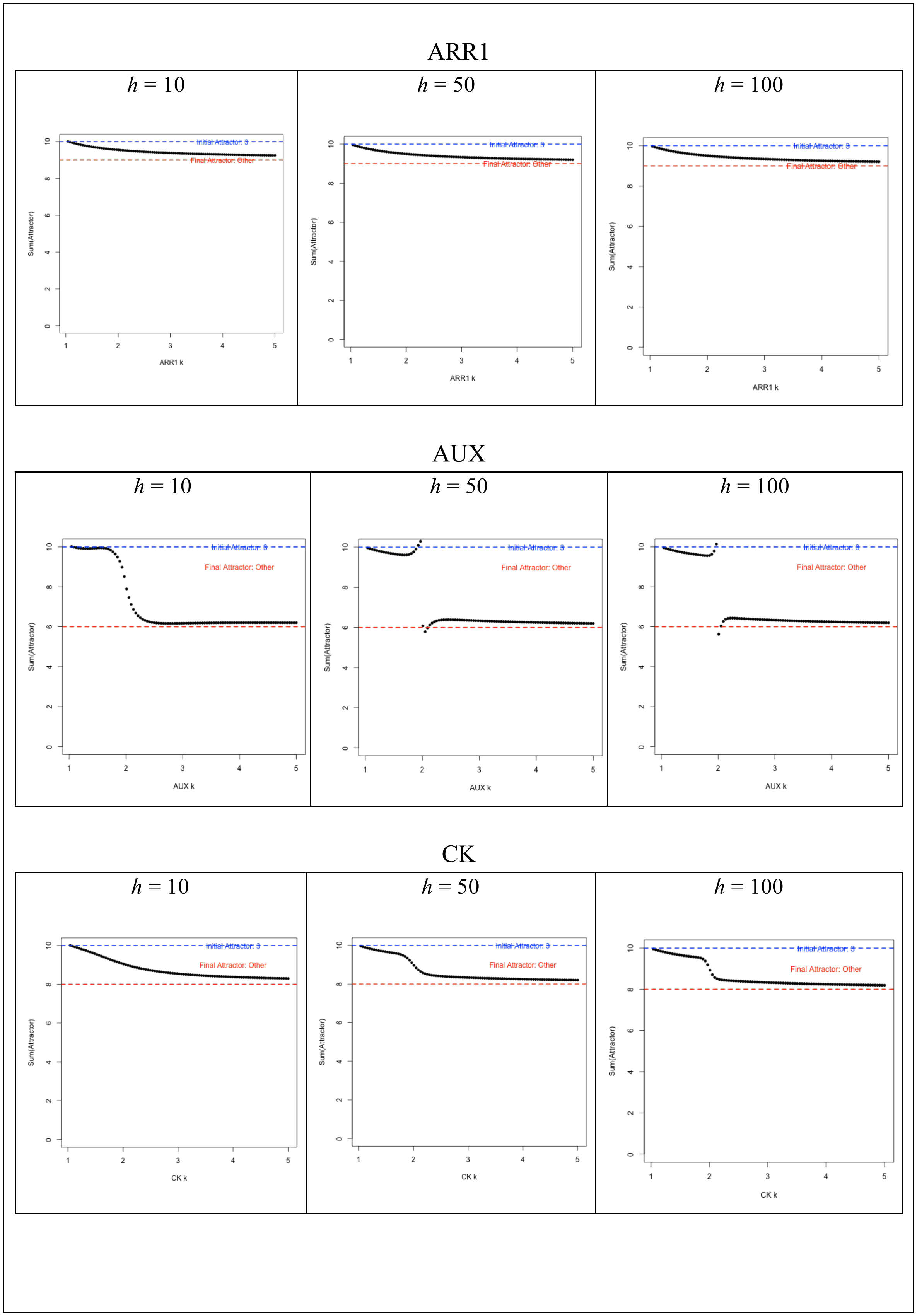
ARR1
h = 10
h = 50
h = 100
Sum(Attractor)
Initial Attractor: 3
Final Attractor: Other
ARR1 k
AUX
h = 10
h = 50
h = 100
Sum(Attractor)
Initial Attractor: 3
Final Attractor: Other
AUX k
CK
h = 10
h = 50
h = 100
Sum(Attractor)
Initial Attractor: 3
Final Attractor: Other
CK k

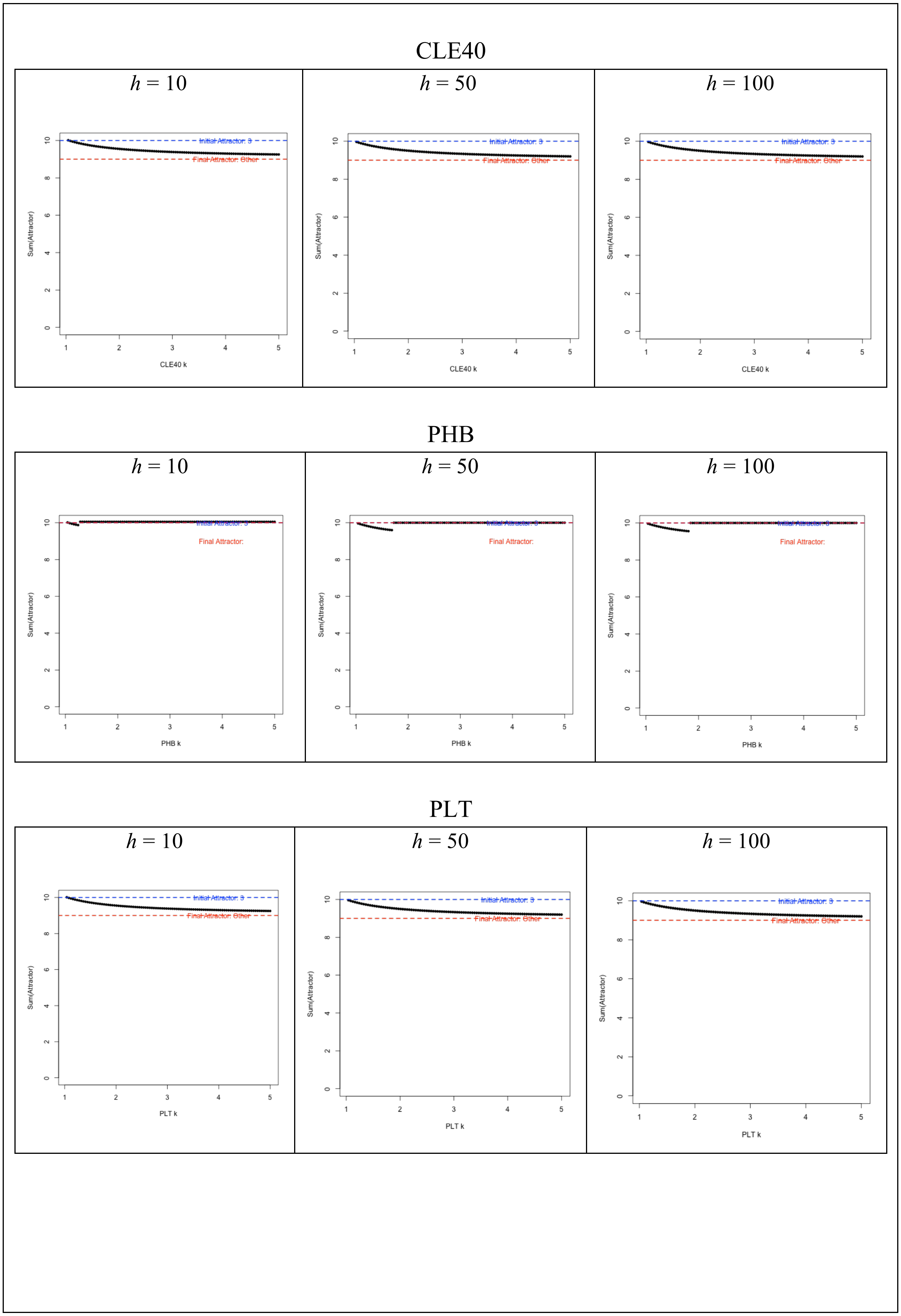
CLE40
h = 10
h = 50
h = 100
Sum(Attractor)
Initial Attractor: 3
Final Attractor: Other
CLE40 k
PHB
h = 10
h = 50
h = 100
Sum(Attractor)
Initial Attractor: 3
Final Attractor:
PHB k
PLT
h = 10
h = 50
h = 100
Sum(Attractor)
Initial Attractor: 3
Final Attractor: Other
PLT k

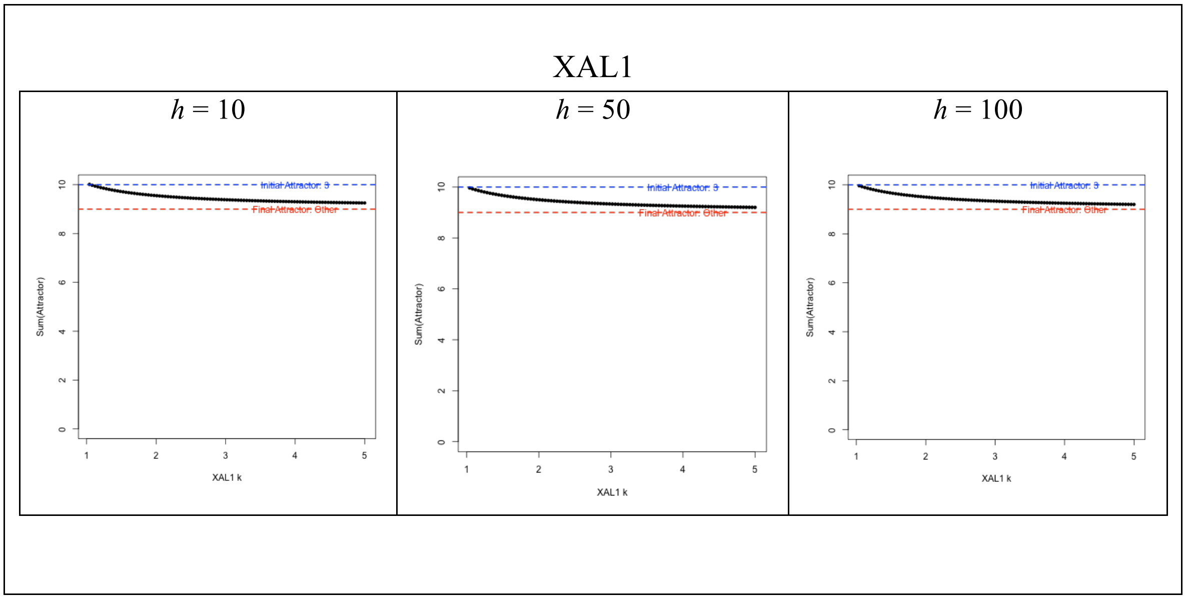
XAL1
h = 10
h = 50
h = 100
Initial Attractor: 3
Final Attractor: Other
Sum(Attractor)
XAL1 k
0
2
4
6
8
10
1
2
3
4
5

E. References

1. Aida, M., Beis, D., Heidstra, R., Willemsen, V., Blilou, I., Galinha, C., ... & Scheres, B. (2004). The PLETHORA genes mediate patterning of the Arabidopsis root stem cell niche. Cell, 119(1), 109-120.
2. Baima, S., Forte, V., Possenti, M., Peñalosa, A., Leoni, G., Salvi, S., ... & Morelli, G. (2014). Negative feedback regulation of auxin signaling by ATHB8/ACL5–BUD2 transcription module. Molecular plant, 7(6), 1006-1025.
3. Burgeff, C., Liljegren, S. J., Tapia-López, R., Yanofsky, M. F., & Alvarez-Buylla, E. R. (2002). MADS-box gene expression in lateral primordia, meristems and differentiated tissues of Arabidopsis thaliana roots. Planta, 214(3), 365-372.
4. Ding, Z., & Friml, J. (2010). Auxin regulates distal stem cell differentiation in Arabidopsis roots. Proceedings of the National Academy of Sciences, 107(26), 12046-12051.
5. García-Cruz, K. V., García-Ponce, B., Garay-Arroyo, A., Sanchez, M. D. L. P., Ugartechea-Chirino, Y., Desvoyes, B., ... & Alvarez-Buylla, E. R. (2016). The MADS-box XAANTAL1 increases proliferation at the Arabidopsis root stem-cell niche and participates in transition to differentiation by regulating cell-cycle components. Annals of botany, 118(4), 787-796.
6. García-Gómez, M. L., Azpeitia, E., & Álvarez-Buylla, E. R. (2017). A dynamic genetic-hormonal regulatory network model explains multiple cellular behaviors of the root apical meristem of Arabidopsis thaliana. PLoS computational biology, 13(4), e1005488.
7. Hao, Y., & Cui, H. (2012). SHORT-ROOT regulates vascular patterning, but not apical meristematic activity in the Arabidopsis root through cytokinin homeostasis. Plant signaling & behavior, 7(3), 314-317.
8. Ioio, R. D., Galinha, C., Fletcher, A. G., Grigg, S. P., Molnar, A., Willemsen, V., ... & Tsiantis, M. (2012). A PHABULOSA/cytokinin feedback loop controls root growth in Arabidopsis. Current Biology, 22(18), 1699-1704.
9. Müller, C. J., Valdés, A. E., Wang, G., Ramachandran, P., Beste, L., Uddenberg, D., & Carlsbecker, A. (2016). PHABULOSA mediates an auxin signaling loop to regulate vascular patterning in Arabidopsis. Plant physiology, 170(2), 956-970.
10. Sabatini, S., Heidstra, R., Wildwater, M., & Scheres, B. (2003). SCARECROW is involved in positioning the stem cell niche in the Arabidopsis root meristem. Genes & development, 17(3), 354-358.
11. Sanchez-Corrales, Y. E., Alvarez-Buylla, E. R., & Mendoza, L. (2010). The Arabidopsis thaliana flower organ specification gene regulatory network determines a robust differentiation process. Journal of theoretical biology, 264(3), 971-983.
12. Santuari, L., Sanchez-Perez, G. F., Luijten, M., Rutjens, B., Terpstra, I., Berke, L., ... & Maeo, K. (2016). The PLETHORA gene regulatory network guides growth and cell differentiation in Arabidopsis roots. The Plant Cell, 28(12), 2937-2951.
13. Shimotohno, A., Heidstra, R., Blilou, I., & Scheres, B. (2018). Root stem cell niche organizer specification by molecular convergence of PLETHORA and SCARECROW transcription factor modules. Genes & development, 32(15-16), 1085-1100.
14. Tapia-López, R., García-Ponce, B., Dubrovsky, J. G., Garay-Arroyo, A., Pérez-Ruíz, R. V., Kim, S. H., ... & Alvarez-Buylla, E. R. (2008). An AGAMOUS-related MADS-box gene, XAL1 (AGL12), regulates root meristem cell proliferation and flowering transition in Arabidopsis. Plant physiology, 146(3), 1182-1192.
